# Dengue Virus Infection of Blood–Brain Barrier Cells: Consequences of Severe Disease

**DOI:** 10.3389/fmicb.2019.01435

**Published:** 2019-06-26

**Authors:** María-Angélica Calderón-Peláez, Myriam L. Velandia-Romero, Leidy Y. Bastidas-Legarda, Edgar O. Beltrán, Sigrid J. Camacho-Ortega, Jaime E. Castellanos

**Affiliations:** Laboratorio de Virología, Vicerrectoría de Investigaciones, Universidad El Bosque, Bogotá, Colombia

**Keywords:** blood–brain barrier cells, breakdown, severe dengue, neurological manifestations, pathogenesis

## Abstract

More than 500 million people worldwide are infected each year by any of the four-dengue virus (DENV) serotypes. The clinical spectrum caused during these infections is wide and some patients may develop neurological alterations during or after the infection, which could be explained by the cryptic neurotropic and neurovirulent features of flaviviruses like DENV. Using *in vivo* and *in vitro* models, researchers have demonstrated that DENV can affect the cells from the blood–brain barrier (BBB) in several ways, which could result in brain tissue damage, neuronal loss, glial activation, tissue inflammation and hemorrhages. The latter suggests that BBB may be compromised during infection; however, it is not clear whether the damage is due to the infection *per se* or to the local and/or systemic inflammatory response established or activated by the BBB cells. Similarly, the kinetics and cascade of events that trigger tissue damage, and the cells that initiate it, are unknown. This review presents evidence of the BBB cell infection with DENV and the response established toward it by these cells; it also describes the consequences of this response on the nervous tissue, compares these evidence with the one reported with neurotropic viruses of the *Flaviviridae* family, and shows the complexity and unpredictability of dengue and the neurological alterations induced by it. Clinical evidence and *in vitro* and *in vivo* models suggest that this virus uses the bloodstream to enter nerve tissue where it infects the different cells of the neurovascular unit. Each of the cell populations respond individually and collectively and control infection and inflammation, in other cases this response exacerbates the damage leaving irreversible sequelae or causing death. This information will allow us to understand more about the complex disease known as dengue, and its impact on a specialized and delicate tissue like is the nervous tissue.

## Introduction

Febrile illness caused by the dengue virus (DENV) is the most important arthropod-borne disease in the world. In the last four decades dengue cases have increased by 30-fold due to the adaptive nature and wide-spread localization of the mosquito vector (Chretien et al., 2015). This increase has also been caused by human migration, chaotic, and rapid urbanization, and deforestation in underdeveloped tropical countries (Arunachalam et al., 2010). Even though dengue is a well-known disease, its clinical manifestations are changing. Nowadays, an increasing number of dengue cases present damage in organs such as brain, liver, heart, or kidneys, an interesting and important feature that has been reported in some cases, but that has been very difficult to relate with the virus given the late onset of these complications. These changes have leaded the World Health Organization (WHO) to include organ damage in the diagnostic criteria for severe cases of dengue (World Health Organization [WHO], 2009). Neurological manifestations, for example, may occur during or after acute infection, may be transient or irreversible (Bopeththa and Ralapanawa, 2017), and may affect both the central (Ng and Sadarangani, 2017) and peripheral nervous systems (Mota et al., 2017). Questions that still need to be answered include: how the DENV enters into the nervous system, where the susceptible cells are located, what kind of cell/tissue responses occur, and what kind of cell/tissue signaling is responsible for nervous tissue damage. It has been proposed that studies on blood–brain barrier (BBB) cells could greatly add to our understanding of the neurotropism, neuro-susceptibility, and neuropathogenesis associated with the DENV.

## DENV Biology and Pathogenesis

Dengue virus belongs to the *Flavivirus* genus which is part of the Flaviviridae family. There are four different serotypes (DENV1 to DENV4) that infect and produce disease in humans. This virus has an icosahedral capsid, a lipoproteic envelope, and a positive, single stranded RNA genome that codes for three structural proteins (C, capsid; M, membrane; and E, envelope) and seven non-structural proteins (NS1, NS2A, NS2B, NS3, NS4A, NS4B, and NS5) (Pierson and Diamond, 2013). The envelope (E) glycoprotein is involved in virus entry into target cells, mainly monocytes, macrophages, hepatocytes, and endothelial cells (EC) using membrane receptors (Grove and Marsh, 2011; Cruz-Oliveira et al., 2015). The non-structural proteins promote RNA translation (NS3) and transcription/replication (NS2B/NS3 and NS5), modulate the innate immune response (NS4A and B) (van Cleef et al., 2013), and play a role in the virion assembly and release (Miller et al., 2007; Pierson and Diamond, 2013).

The epidemic cycle is human–mosquito–human, and the main vectors are the *Aedes aegypti* and *Aedes albopictus* females (Bhatt et al., 2013). After a mosquito bite, the inoculated saliva/virus is transported via skin dendritic cells toward the lymphatic nodes where it infects monocytes, replicates, and spreads to the blood and other organs (Pierson and Diamond, 2013). Most infected people do not have symptoms (Endy et al., 2011; Castellanos J. et al., 2016), but others develop subtle symptoms such as fever and malaise. Some patients present muscle and bone pain, high fever, ocular headache, abdominal pain, thrombocytopenia, and mucosal bleeding. This is classified as dengue with warning signs. Around 10% of patients may develop severe signs and symptoms include severe hemorrhages, massive plasma leakage with edema, and severe organ dysfunction. This is diagnosed as severe dengue which may lead to death (World Health Organization [WHO], 2009).

In addition to clinical signs and symptoms, a case confirmation is necessary and should be determined through serology or molecular tests. However, the sensibility and specificity of tests depends on which day of illness they are undertaken (Guzman and Harris, 2015). Unfortunately, neither clinical following nor laboratory tests allows for the accurate prediction of the outcome in each case.

Disease severity is explained by different factors such as age, nutrition status, genetic background (Guzman et al., 2002; Sierra et al., 2007; Guzman and Harris, 2015), and the infecting virus serotype or genotype (Cologna et al., 2005; Martina et al., 2009). The most prominent factor which leads to severe dengue fever with complications is having a second or third infection with a different DENV serotype (Descloux et al., 2009; Ohainle et al., 2011; Guzman and Harris, 2015). However, a role for NS1 protein has been described, for example activating the complement system or eroding EC glycocalyx (Puerta-Guardo et al., 2016).

The immune response during infection activates monocytes and macrophages (Puerta-Guardo et al., 2013), dendritic cells (Luplertlop et al., 2006), T cells (Lühn et al., 2007), and mastocytes (St John et al., 2013) which release large amounts of cytokines and chemokines to affect, for example, EC (Avirutnan et al., 2006; Trung and Wills, 2010). Synergic immune cell responses lead to cytokine storms, plasma leakage, and organ failure. Therefore, dengue severity depends on the individual response to infection, which has several types of clinical presentations and, frequently, has no correlation with immune markers; this makes accurate diagnosis or prognosis very difficult.

## Dengue With Neurological Manifestations

As stated above, most DENV infections are asymptomatic or mild, but in less likely cases with severe presentation, signs can begin either early or late and involve different systems and organs such as gastrointestinal, hepatic, renal, cardiovascular, respiratory, musculoskeletal, and neurological (Gulati and Maheshwari, 2007; Puccioni-Sohler et al., 2012). Symptoms and signs in the nervous system have been published since 1976 (Sanguansermsri et al., 1976), however, recently there has been an increase in case reports, experimental *in vivo* and *in vitro* research, meta-analysis, and reviews which show great concerns about these signs and the change in the clinical picture over the last few years.

Neurological alterations occur in both males and females and in every age group in endemic areas. Any of the four DENV serotypes may be involved, although DENV2 and DENV3 are the most frequently identified by RT-PCR or viral isolation (Hapuarachchi et al., 2015). Nevertheless, neurological manifestations as consequence of DENV infection are still reported as infrequent and given the diversity of clinical manifestations, neurological signs can be quite subtle (Wilder-Smith et al., 2019). Nervous system alterations may or may not be reversible and may take several days or months for complete recovery, as an example, Table 1 summarizes some of the most interesting clinical reports of patients that presented neurological manifestations after or during DENV (Table 1), or other flavivirus (Supplementary Table S1) infection.

**Table 1:** Clinical reports of dengue cases with neurological involvement.

DENV								
**Patients/age (years)**	**Viral symptoms**	**Neurologic symptoms**	**(+) Lab tests**		**Molecular and other tests**	**Dx**	**Outcome (number of cases)**	**References**
							
			**Serum**	**CSF**				

55 CH (0.1–12); F (14); M (9–67)	Nausea, headache, vomiting, rigors, fever, rash, myalgia.	Confusion, seizures, unusual behavior, focal neurological signs, fluctuating consciousness, somnolence.	IgM NS1 IgG	IgM	**CT:** Edema and ischemic infarcts. **MRI:** Restricted diffusion. **EEG**: Encephalitis **PNRT** and **IMH:** DENV (+). **BTB:** Cerebral edema.	ENC ENP	L (12) RWS (2) CR (8)	Lum et al., 1996; Angibaud et al., 2001; Nogueira et al., 2002; Chotmongkol and Sawanyawisuth, 2004; Méndez and González, 2006; Rao et al., 2013; Mathew et al., 2014; Fong et al., 2017
M (17; 25) F (27)	Fever, myalgia, arthralgia, vomiting, headache.	Seizures, coma, stiffness, amnesia, memory impairment	IgM	IgM IgG	**BTB:** Infection of N, A, M and EC. **MRI:** Subdural hematoma	HVS MI AP	L (1) CR (1) RWS (1)	Ramos et al., 1998; Yeo et al., 2005; Mittal and Jain, 2011
F (17; 28) M (42)	Headache, fever, vomiting, chills, diarrhea.	Confusion, K/B test (+), seizures, (↓) consciousness, fixed pupils, no motor response.	IgM NS1 IgG	IgM IgG	**RT-PCR**: DENV (+)	DF SD	CR (2) L (1)	Goswami et al., 2012; Hapuarachchi et al., 2013
M (41), F (45)	Headache, myalgia, arthralgia, fever, chills	Loss of balance	IgM IgG NS1		**TEM:** VLP in brain parenchyma. **NE:** Lack of coordination, ataxic gait	HVS AC	L (1) CR (1)	Limonta et al., 2012; Withana et al., 2014
M (18)	Fever, myalgia, headache, lethargy	Loss of balance, speech difficulty, weakness.	IgM IgG NS1		**NE:** Slight tremors, tongue in right protrusion	PMN/CA/BP	RWS	Azmin et al., 2013
F (13, 24)	Fever, arthralgia, myalgia, headache	Seizures, rigidity in extremities.	IgM IgG	IgM IgG	**MRI:** Hemorrhages **CT:** Brain stem herniation	HENC SD	RWS (1) L (1)	Nadarajah et al., 2015
F (12; 39)	Fever, myalgia, nausea, diarrhea, chills	Weakness, flaccid quadriplegia, autonomic instability, involuntary muscle tightening and twitching	IgM IgG		**MRI:** Epidural hematoma. **TMBS:** Motor potentials absent.	TM Mild NMC	RWS (1), CR (1)	Fong et al., 2016; Doi et al., 2017


*Summary of clinical reports of patients with neurological consequences during or after DENV infection. AC, acute cerebellitis; AP, axonal polyneuropathy; BP, brachial plexopathy; BTB, brain tissue biopsy; CA, cerebellar ataxia; CH, children; CR, complete recovery; CT, computed tomography; DF, dengue fever; Dx, diagnosis; EEG, electroencephalogram; ENC, encephalitis; ENP, encephalopathy; F, female; HVS, hypovolemic shock; HENC, hemorrhagic encephalitis; IMH, immunohistochemical analysis; K/B, Kernig and Brudzinski test; L, lethal; M, male; MI, memory impairment; MRI, magnetic resonance imaging; NE, neurological exam; NMC, neuromuscular complications; PMN, Parkinsonism with multiple cranial neuropathies; PRNT, plaque reduction neutralization test; RWS, recovered with sequelae; RT-PCR, polymerase chain reaction with retro-transcription; SD, severe dengue; TEM, transmission electron microscopy; TM, transverse myelitis; TMBS, transcranial magnetic brain stimulation; VLPs, viral like-particles; (+), positive; (-), negative; (↓), decrease.*

The nervous alterations could be classified following certain criteria, such as: (i) direct tissue infection (encephalitis, meningitis, myositis, myelitis, rhabdomyolysis; (ii), signs related with systemic or metabolic imbalance (encephalopathy, stroke) and (iii) early or late post-infection sequelae (transverse myelitis, Guillain-Barré syndrome – GBS-, acute disseminated encephalomyelitis) (Murthy, 2010). A detailed description of neurological complications during dengue infection was recently published (Li et al., 2017).

According to this evidence, nervous tissue damage could be caused directly by the virus and/or the immune response, which was originally established to get rid of the virus. However, it is not clear how the virus enters the central nervous system (CNS), which cells are infected and respond, and how equilibrium is reached (or not) after injury. So far, the most probable DENV entry route to the CNS is the hematogenous route (Lanteri and Busch, 2012; Velandia and Castellanos, 2012; Guzman and Harris, 2015), therefore, the BBB plays an important role in favoring the neuroinvasion and neurotropism of the virus. Alteration in its functioning can help to understand the morphological and physiological consequences of the arrival of the virus to the brain. These aspects and the evidence on BBB participation in flavivirus neurological findings have been presented in this document.

## Blood–Brain Barrier (BBB) Characteristics

Brain capillary structures carry oxygen and nutrients to nervous cells (Obermeier et al., 2013). EC lining these vessels have a primary role in permeability regulation and with other cells, form highly organized barriers between the vascular space and nervous tissue parenchyma (Zhao et al., 2015). The neurovascular unit (NVU) consists of the aforementioned capillary EC, pericytes, astrocytes, microglial cells, and neurons joined by close direct and indirect interactions that maintain and regulate brain homeostasis (Abbott et al., 2006; Cardoso et al., 2010). Despite the high selectivity for solutes and molecules, sometimes bacteria, parasites, and viruses can pass through the BBB and invade nervous tissue (Zhang et al., 2015); in these cases, the local or systemic immune response contributes to altering NVU integrity (Hou et al., 2016).

Some viruses can reach the nervous parenchyma in different ways: (i) entering of infected leukocytes; (ii) axonal transport; and (iii) olfactory bulb epithelium infection. Other virus entry involves EC, (iv) inter-endothelial tight junction disruption and (v) EC infection and basolateral release (Miner and Diamond, 2016; Tohidpour et al., 2017). The latter two ways as well as the passing through of infected monocytes may be the most critical routes used by DENV to reach the CNS. For example, Hapuarachchi et al. (2015) reported a fatal case due to DENV4 infection. Importantly, the patient presented an accelerated neurological damage and although the virus was detected in serum, the viral titer was higher in cerebrospinal fluid (CSF), suggesting a rapid virus entry through BBB (Hapuarachchi et al., 2015). Below, we present the *in vivo* and *in vitro* evidence on the single participation of each of the NVU cells and the whole structure participation in CNS infection by DENV. We also recapitulate evidence of BBB cells infection by other important flaviviruses (Supplementary Tables S2, S3).

## Endothelial Cells

Plasma leakage is the central phenomenon in severe dengue (SD) cases. It is caused either by EC homeostasis alteration due to direct DENV infection or because EC are a target of inflammatory mediators secreted by either them or by other infected cells (Avirutnan et al., 1998). The first report of EC infection due to DENV used serotype 2 and primary cultures of rabbit cava vein and human umbilical vein EC (Andrews et al., 1978). Despite the existence of many studies, until recently the molecular events and specific receptors involved in DENV binding and entry to EC were unclear (Yang et al., 2016).

Dengue virus entry can occur in a receptor-mediated binding with or without clathrin participation manner, but in immune cells it also uses virion-IgG complex to bind Fc-gamma receptors to infect these cells (Wei et al., 2003; van der Schaar et al., 2008). This could also be a mechanism used in EC (Cruz-Oliveira et al., 2015).

Regarding the latter, using a virus overlay protein binding assay (VOPBA) in ECV304 cells, it was possible to identify three new cell membrane proteins of 29, 34, and 43 kDa, which binds to a recombinant E protein, suggesting that these proteins may be important for DENV entry and internalization in these cells (Wei et al., 2003).

Later, integrin β3 was also identified as an EC receptor to DENV, since protein knockdown reduced viral entry by approximately 90% (Zhang et al., 2007). Similarly, HMEC-1 cells upregulate protein disulfide isomerase (PDI) during infection. Silencing its expression reduces the number of infected cells and the production of new virions (Wan et al., 2012). Recently, it was shown that the Rod domain of the intermediate filament vimentin, which is exposed at the EC surface, may interact with domain III of the DENV envelope protein. This indicates a role in the binding of the virus to the endothelium (Yang et al., 2016).

Additional evidence shows that DENV binding to EC induces Rho-associated coiled-coil-containing kinase (ROCK) activation and vimentin reorganization, which induces endoplasmic reticulum redistribution for more efficient virus replication and assembly (Lei et al., 2013). Membrane sugars and proteoglycans, such as heparan sulfate proteoglycan, act as virus-binding molecules, given that treatment with heparin or heparan-, chondroitin-, and dermatan-sulfate inhibited the infection up to 60% (Dalrymple and Mackow, 2011). Finally, evidence exists of the following molecules as EC receptors to DENV: DC-SIGN, ICAM-3, CD14, mannose receptor (CD206), heat shock proteins (HSP70 and HSP90), glucose-regulated protein (GRP78), and laminin receptor (Dalrymple and Mackow, 2014). However, their direct participation remains to be investigated.

Although, *in vitro* it is possible to evaluate and quantify DENV infection in EC, infection and replication is often not detected *in vivo.* In some cases, antigens or viral RNA have been detected in EC from brain biopsies of dengue fatal cases -particularly EC’s from the inferior olive nucleus, medulla (Calvert et al., 2015) and cerebellar granular layer (Ramos et al., 1998), other reports, had shown viral RNA or proteins in EC from liver and lung (Jessie et al., 2004; Póvoa et al., 2014), suggesting viral replication in these cells (Basu and Chaturvedi, 2008).

There is no consensus about the effect of DENV on EC. Some reports have found no morphological changes in EC after infection (Srikiatkhachorn and Kelley, 2014). However, apoptotic EC were detected in the lungs, guts, and brains of Cuban patients who died due to DENV infection (Limonta et al., 2007). In another study, ECV340 cells infected with DENV2 had a 7–40% increase in the number of apoptotic cells at 48 h post infection (p.i.) (Avirutnan et al., 1998). A similar result was reported after using an HMEC-1 line after infection with a DENV2 clinical isolate. Apoptosis was detected and activated by the cleavage of caspase 3 and poly (ADP-ribose) polymerase 1 (PARP-1) by the viral protease complex NS2B/NS3 (Vásquez et al., 2009).

Rodents are not naturally susceptible to DENV infection, delaying the implementation of suitable animal models to study infection and pathogenesis (Yauch and Sheresta, 2008). Viremia and clinical signs in non-human primates are mild (Zompi and Harris, 2012). This leads to the use of immunodeficient, humanized, or modified mice (Wu-Hsieh et al., 2009; Plummer and Shresta, 2014), or the production of adapted virus to specific hosts, with the purpose of implement models to study the disease, antiviral, or vaccine development (Puccioni-Sohler and Rosadas, 2015). These infection models have advantages and flaws but are frequently used to better understand dengue disease (Srikiatkhachorn and Kelley, 2014).

Using mouse models, the nervous tissue infection has been evaluated. A previous work reported BBB damage in Balb/c mice inoculated intracerebrally (i.c.) with DENV2. Researchers found Evans blue leakage and radiolabeled erythrocytes in brain parenchyma, a phenomenon explained by the increase of cytokines (Chaturvedi et al., 1991). Recently, we established a DENV neuroinfection model inoculating the neuroadapted D4MB-6 strain intraperitoneally (i.p.) in 2- and 7-day-old Balb/c mice. This adapted strain (DENV4 mouse brain passaged six times) reaches the CNS and induces severe brain cytoarchitectural changes, leukocyte infiltration, and BBB dysfunction associated with EC activation (Velandia-Romero et al., 2012). A similar finding in adult mice was reported recently, and viral replication occurred in EC from the cortex, hippocampus, and cerebellum detected by the NS3 expression, concomitant with BBB disruption (Solomão et al., 2018). In this case, the DENV2 was isolated from a patient with confirmed encephalitis, suggesting that natural DENV strains are neurotropic and neurovirulent, able to evade the immune system, and invade the brain efficiently through BBB ECs, leading to replication in the brain parenchyma which induces nervous injury.

Dengue virus-infected EC are low in most *in vitro* experiments using either primary cultures or cell lines (Calvert et al., 2015). The proportion of DENV-4-infected mouse brain microvascular EC (MBEC) cultured in our laboratory were 7 and 12% at 24 and 48 h p.i. respectively, but the neuroadapted D4MB-6 strain infected half of the cells at 48 h p.i. (Velandia-Romero et al., 2016). These differences between models may be explained by the presence of heparin in some media cultures for EC which may inhibit the infection. Infection percentages also depend on DENV strain or the concentration of NS1 in the inoculation media, since this protein forms complexes with HSPG competitively, inhibiting the EC infection (Calvert et al., 2015). In fatal cases, the likelihood of finding viral proteins or RNA depends on the time of tissue collection, because in later periods the virus may be cleared making detection impossible (Srikiatkhachorn and Kelley, 2014).

The apparent low susceptibility of EC to DENV infection observed in post-mortem samples contrasts with the profound effect that the infection has on these cells, in particular on the BBB. This suggests that other factors, such as inflammatory molecules, amplify the effects of DENV and alter the endothelial tissue and other cells of the NVU (Calvert et al., 2015).

Endothelial cell activation due to infection was reported based on the expression or secretion of sVCAM-1 and sICAM-1 (Cardier et al., 2006), which has also been detected in the membrane of HMEC-1 and LSEC (Peyrefitte et al., 2006). The D4MB-6 neuroadapted strain after the infection of MBEC also induced VCAM-1 and ICAM-1 expression, as well as PECAM-1 and E-selectin (Velandia-Romero et al., 2016). Interestingly, the immune mediators secreted by infected EC depends on the serotype used, i.e., DENV1 induced interleukin (IL) -6, TNF-α, chemokine (C-X-C motif), ligand 1 (CXCL1), CCL2, CCL5, and CCL20 (Soe et al., 2017). All of these molecules are associated with endothelial hyperpermeability and change the coagulatory balance related to hemorrhages and disseminated intravascular coagulation seen during severe dengue (Srikiatkhachorn and Kelley, 2014). However, DENV2 infection induced monocyte chemoattractant protein 1 (MCP-1), matrix metalloproteinases (MMP), and macrophage migration inhibitory factor – MIF – (Lima-Juniora et al., 2013).

## Pericytes

These cells surround the EC and are immersed in the basal membrane of microvessels and capillaries. Pericytes have a crucial role in the maturation and function of the BBB since they interact with both EC and astrocytes inside the NVU (Yamakazi and Mukouyama, 2018). To our knowledge, there have been no reports on DENV infection or susceptibility to it of pericytes, although Japanese encephalitis virus (JEV), another *Flavivirus*, is known for infecting them and affecting the BBB function.

Japanese encephalitis virus is responsible for most encephalitis cases in Asia, with around 68,000 cases reported each year, 30% of which result in fatal outcomes (Thongtan et al., 2012). This virus was isolated for first time in 1935 (Solomon et al., 2003; Mishra et al., 2008) and is transmitted via *Culex* spp. mosquitoes. Fever, headaches, meningeal irritation, and an altered state of consciousness are the primary signs and symptoms (Thongtan et al., 2010). During infection, 30–50% of patients may present paralysis, loss of speech, and behavioral changes (Chen et al., 2000; Thongtan et al., 2012).

Several molecules have been proposed as cell receptors to JEV, including HSPG, HSP70, and HSP90B, vimentin, CD4, laminin receptor, and α5β3 integrin. However, the pericyte molecule involved in the infection is not yet known (Nain et al., 2016).

Even though EC obtained from brain microvascular vessels are susceptible to JEV, the *in vivo* BBB function and integrity is not affected during infection. However, EC cultures exposed to JEV-infected pericyte supernatants experienced an increase in permeability and loss of *trans*-endothelial electrical resistance (TEER). These changes in function were related to the presence of IL-6, MMP-2 and -9, and the proteasomal degradation of the zonula occludens protein – ZO-1 – (Chen et al., 2014), which is an important scaffold protein, whose interaction with others tight junctions proteins (TJP) and the actin filaments of the EC are crucial for the maintenance of endothelial barrier function and the regulation of vascular permeability (Garcia-Ponce et al., 2015). Together, these findings suggest that the immune response of the pericytes play a central role in endothelial dysfunction during JEV infection (Chang et al., 2017).

Recently, it was reported that the *Flavivirus* ZIKV infects retinal pericytes and retinal EC in addition to pigmented epithelium cells (Roach and Alcendor, 2017). Interestingly, pericytes replicate ZIKV more efficiently than EC, but they do not die during infection. Both cell types, after 4 days p.i., secreted high levels of RANTES, a chemokine that favors the retina inflammation and ocular injury that occurs in ZIKV infection (Roach and Alcendor, 2017). In this work, it is suggested that the virus enters in a hematogenous way, through EC from retinal arteries and capillaries. This is because both pericytes and EC are susceptible to infection and able to replicate the virus, allowing it to spread to other eye cells. These findings suggest that pericytes may play a role in BBB, permitting the infection and secreting the immune mediators that modify the barrier physiology.

## Astrocytes

These cells are the main components in the architecture and function of the CNS. In addition, to providing metabolic support to neurons, astrocytes can also regulate neuronal activation and function (Abbott et al., 2006), as they store information and regulate the synaptic transmission participating in the tripartite synapsis with pre- and post-synaptic neurons (Perea et al., 2009). Astrocytes modify the EC and BBB function (McConnell et al., 2017) due to their permanent contribution to nervous system defense and recovery after an infection, or physical or metabolic damage (Zorec et al., 2018). For example, astrocytes express pathogen-associated molecular patterns (PAMP) and Toll receptors (Krasowska-Zoladek et al., 2007; Suh et al., 2009) that recognize bacteria and viruses (Kigerl et al., 2014) in addition to MHC-I and II (Dong and Benveniste, 2001) to recognize and present antigens. These cells also secrete at least 200 different molecules immunomodulators, hormones, and growth factors (Verkhratsky et al., 2016). This means that after a stimulus they respond with cell activation (astrogliosis or astrocytosis) and promote neuroinflammation. There are few studies on DENV infection effect on astrocytes, and those that are available provide contradictory evidence. For example, Balb/c astrocytes (Type I and II) were exposed to three different strains of DENV2 and one of DENV4 and no infection was reported after 15 days p.i. (Imbert et al., 1994). Similarly, GFAP positive astrocytes were not infected after the i.p. inoculation of the neuroadapted D4MB-6 dengue strain (Velandia-Romero et al., 2012), and neither viral protein nor RNA were detected in astrocyte cultures at 48 h p.i. (Velandia-Romero et al., 2016). Despite this, *in vitro* astrogliosis post viral inoculation was confirmed evaluating the cells morphological changes.

Astrocytes infection with DENV has been reported in fatal human biopsy cases (Bhoopat et al., 1996), like the one reported in Mexico (Ramos et al., 1998). However, in every case, regardless of whether the astrocytes were infected or not, researchers reported the early and robust response to infection as astrogliosis, which was maintained even after nervous tissue viral clearance. Therefore, astrogliosis could be involved in long-term complications presented in some patients (Lee et al., 2016).

During the activation process, astrocytes change function, morphology, and biochemistry (Middeldorp and Hol, 2011), induced by local signals that depend on the localization and duration of the harmful stimulus or aggression (White and Jakeman, 2008). Astrocytes in injured tissue secrete pro-inflammatory molecules such as IL-6, TNF-α, and interferon β (Boonnak et al., 2011; Burkert et al., 2012).

Significant astrogliosis and progressive cell hypertrophy with longer and thicker astrocytic processes may be induced by strong or continuous stimuli (Eng et al., 2000). These morphological changes are associated with the overexpression of cytoskeleton proteins such as GFAP, vimentin, and nestin (Buffo et al., 2010), which are essential for cell proliferation process and glial scar formation (Sofroniew, 2009).

Rhesus macaques inoculated with intravenous or subcutaneous injections of DENV2 experienced a reduction in the number of astrocytes in the frontal lobe compared to non-infected control monkeys. However, cytoplasmic enlargement and an increase in the number and length of astrocytic end-feet in white matter were found (Lee et al., 2016). Generalized astrogliosis was seen in 2-, 7-, and 14-day-old Balb/c mice infected with the neuroadapted strain D4MB-6, finding large numbers of GFAP+ cells that were larger in size, more branched, and with greater fluorescence intensity. The latter results were related with the evidence of paralysis, which is a sign of neural impairment along with the histopathological evidence of neuron infection and efficient viral replication in these cells. Interestingly, the older mice (14 days old) were refractory to infection and showed no neurological symptoms. Nevertheless, they had astrocytic activation with the same changes, demonstrating that the immune and physiological development of these individuals determines their susceptibility to DENV infection, favoring brain virus clearance (Velandia-Romero et al., 2012).

On the other hand, it has been shown that astrocytes are susceptible to the West Nile Virus (WNV) and that they can become viral reservoirs because WNV persists for up to 114 days after infection in astrocytes without inducing cell death (Diniz et al., 2006). This virus shares some characteristics with DENV and JEV and currently is a concern in many countries experiencing permanent outbreaks (Solomon and Mallewa, 2001). In addition to infection in Langerhan’s cells and keratinocytes, after the initial replication, WNV infects neurons, astrocytes, microglial cells, and EC, leading to a massive recruitment of inflammatory cells toward the brain parenchyma (Hussmann and Fredericksen, 2014; Winkelmann et al., 2016). Clinically, the infection is characterized by meningitis, encephalitis, and acute flaccid paralysis. In severe cases it can cause the death of the patient (Hussmann and Fredericksen, 2014).

Another factor to consider during infection and glial activation is the brain lymphatic system (BLS) (Louveau et al., 2015; Sandrone et al., 2019), represented by the classic lymphatic vessels in the dura mater, and the canals formed by the walls of the veins and astrocytic endfeet (called the glymphatic system) (Iliff et al., 2012; Yang et al., 2013; Louveau et al., 2015). Importantly, the function of this structure is to drain debris and interstitial macromolecules from the brain (Aspelund et al., 2015). Additionally, BLS allows the circulation of T lymphocytes, which in response to infection can be protective or pathogenic (Louveau et al., 2015).

In fact, the drainage of viral antigens from the parenchyma to the CSF (made by the glymphatic system), and from there to the lymphatic vessels, can contribute to suppression or control of the T cells response in the periphery (McGavern and Kang, 2011; Louveau et al., 2015). This would explain the tolerance and little activity of the extra neural immune system during some nervous system infections with virus like HIV (Tan et al., 2012), MCMV (Torti et al., 2011) and parasites like *Toxoplasma gondii* (Landrith et al., 2015). Interestingly, although it is known that the lymphatic system is supremely important during the dispersion of DENV in the periphery (Yam-Puc et al., 2016), its participation in the dispersion of the virus within the nervous system by recirculating the virus that might be present in the CSF is not known or evaluated. Similarly, its role in the control of infection and its contribution to the evasion of the response within nervous tissue is unknown.

## Microglial Cells

According to their mesodermal origin, microglial cells are immune cells of the CNS (Ginhoux and Prinz, 2015) which express macrophage markers such as F4/80, Fc receptors, and CD11b. During brain injury, microglial cells become reactive and secrete inflammatory mediators similar to those produced by macrophages (Lannes et al., 2017). Microglia establishes bidirectional communication with neurons, astrocytes and pericytes, which is a determining factor during development and brain function in adults. In pathological conditions, an early reaction of microglia inducing and regulating the astrocytes function occurs (Jha et al., 2018). These cells are susceptible to DENV, possibly due to the expression of previously reported viral receptors such as, HSPG, CD14, DC-SIGN, GRP78, HSP70 and HSP90, β3 integrin, mannose receptor, and C-type lectin domain containing 5A (CLEC5a), and others (Thongtan et al., 2012), just like it has been described in the murine microglial cell line BV2, previously reported as susceptible to all four DENV serotypes (Bhatt et al., 2015).

After binding to a receptor, clathrin-mediated endocytosis is used to internalize the DENV (Jhan et al., 2017) and start replication, an event that triggers cell activation, leading the morphological change from round cells to multipolar cells, and the secretion of inflammatory molecules. These changes can be observed *in vitro* in BV2 cells (Bhatt et al., 2015), and in the brain of young mice infected with neuroadapted D4MB-6 strain, where infected microglial cells were also observed frequently in the analyzed tissue (Puccioni-Sohler and Rosadas, 2015). Interestingly, each DENV serotype infection induces different responses in BV2 microglial cells. DENV1 induces a cytokine profile related with changes in vascular permeability, while DENV2 alters the oxidative stress-mediated apoptotic response, and DENV3 establishes an anti-inflammatory and antiviral mediators profile. On the other hand, mediators induced by DENV4 could have a significant role in the alteration of BBB function, mainly by enhancing the secretion of MMP (Bhatt et al., 2015). Infection with DENV2 and DENV4 caused the activation of rat microglial cells (HAPI), but serotype 4 induced a higher ROS production and greater cell death compared to DENV2 or even JEV (Suwanprinya et al., 2017), suggesting a noxious role in brain inflammation and neurotoxicity.

The response of microglia may be essential to protect DENV-infected mice, as the animals treated for macrophage/microglia depletion with clodronate liposomes showed higher viral RNA and protein expression as well as more severe neurological signs. The reduction of microglial cells in the brain was also related with lower IFN-γ and IL-12 secretion and less infiltrated TL CD8 cells (Tsai et al., 2016). Taken together, these results support that, despite microglial cells susceptibility to DENV infection, their response contributes to delay nervous tissue damage, and like it happens during infections with JEV, microglial cells may play the role of Trojan horse, transporting the virus and serving as viral reservoirs for up to 16 weeks (Nain et al., 2016).

## Glial Activation

Microglial and astrocytic cells are the essential pillars of the immune response during a viral infection in the CNS (Ramesh et al., 2013; Bhatt et al., 2015). Overall, after the pathogen is recognized, astrocytes and microglial cells trigger two types of events: (i) an activation of the innate immune response in neighbor cells, amplifying the signal, and (ii) a favoring of the changes in BBB permeability, contributing to blood leukocyte migration to the brain parenchyma, and helping in the adaptive immune response (Jhan et al., 2017). As part of the response, mediators such as IL-6, IFN-γ, GM-CSF, IL-1, and TNF-α are secreted. These induce changes in BBB permeability and together are the necessary stimuli to differentiate and induce proliferation of monocytes, astrocytes, microglial cells, and EC (van Kralingen et al., 2013). The production of chemokines and the induction of adhesion molecule expression facilitate T cells entering and surviving, and the proliferation of B cells in the tissue (van Kralingen et al., 2013). As part of a general response, during infection, astrocytes and microglial cells secrete anti-inflammatory and neuroprotector molecules that induce suppression and neural protection. The expression of co-stimulatory molecules such as B7 and B40 of the adaptive immune response (Farina et al., 2007) is a key mediator to diminish the damage in nervous tissue (Miljkovic et al., 2007).

## Neurons

In addition to their role in information processing, neurons contribute to regulating the other nervous system cell functions. These cells secrete VEGF and establish physical contact with NVU cells during development to regulate their activity and stimulate BBB growth and branching (Ruhrberg and Bautch, 2013). Once the vascular network is established, the cytoplasmic processes of neurons and astrocytes control the vessels lumen diameter, releasing vasoactive substances such as nitric oxide (Gotoh et al., 2001), acetylcholine (Scremin et al., 1973), neuropeptide Y (Abounader et al., 1995), and somatostatin (Cauli et al., 2004; McConnell et al., 2017), demonstrating the principal role of neurons in BBB homeostasis.

Peripheral DENV infection and replication induces a “cytokine storm” which affects many tissues and even modifies brain homeostasis and neurological signs without direct infection. Neuronal infection has been detected in tissues obtained from fatal cases (Ramos et al., 1998; Nogueira et al., 2002; Guzman and Harris, 2015), however, there have been documented cases of infections and neurological alterations such as loss of consciousness, sensory or motor dysfunction and analgesia in the early post-infection times (Shweta et al., 2014).

Animal models of dengue infection have shown neuronal infection and nervous tissue injury (Hotta et al., 1981). One of the first works describing viral antigens in neurons after intracerebral inoculation also detected proteins of the virus in neuronal axons. Ultrastructural descriptions showed neuronal necrosis and nuclear clefts (Sriurairatna et al., 1973).

Approximately 40% of primary cultured Balb/c neurons were infected using a MOI 5 (Salazar et al., 2013) and experiments in the mouse cell line Neuro-2a showed viral internalization after 2 h post inoculation. The virus was detected using a plaque assay after 24 h p.i. (Ho et al., 2017), reinforcing the evidence for some DENV neurotropism, although the neuronal molecules responsible for the infection promotion remain unknown. Using VOPBA experiments there has been identified a 65 kDa protein which binds specifically to DENV (Ramos-Castañeda et al., 1997). Interestingly, *in vivo* and *in vitro* dopamine receptor-blocking induced a reduction of neurological symptoms in mice and diminished the number of infected neurons (Simanjuntak et al., 2014). A similar finding was observed in Neuro-2a cells (expressing D2R) and mouse hippocampal neurons (expressing D1, D2, and D5 receptors) since treatment with metoclopramide reduces the infection, suggesting the participation of dopaminergic receptors in DENV infection (Ho et al., 2017). This work also demonstrated that Neuro-2a cells treated with an inhibitor of clathrin-mediated endocytosis (chlorpromazine) or an inhibitor of clathrin-independent endocytosis (Pitstop-2) induces a significant infection reduction, replication, and virus release, indicating the participation of both endocytosis systems (Hou et al., 2016), such as those previously reported by van der Schaar et al. (2008).

Despite many authors proposed that DENV should not be considered as a neurotropic or neurovirulent flavivirus, there is evidence of CNS entry by hematogenous and axonal transport routes (Chaturvedi et al., 1991; Mc Minn, 1997). However, the DENV neurotropism and neuroinvasion determinants are still little known (Velandia-Romero et al., 2012). A monocyte-released free virus could infect EC in brain capillaries and reach the parenchyma by passive diffusion or transcytosis (Mc Minn, 1997; Liou and Hsu, 1998; Chantick et al., 2016) and infect neurons and microglial cells, like occurs with other *Flaviviruses* (Ludlow et al., 2016). However, axonal transport used by many neurotropic viruses, such as herpes, rabies, JEV, and WNV, may travel inside the axons as nude capsids or in vesicles until they reach the neuronal soma in the brain and spread to other areas using the axonal transport (Hunsperger and Roehrig, 2009; Salinas et al., 2010; Ludlow et al., 2016).

Immunodeficient neonatal mice inoculated with DENV2 showed viral particles in both myelinic and amyelinic fibers of pre- and post-synaptic neurons by electronic microscopy at 5 days p.i., suggesting an axonal spreading of DENV in the brain and spinal cord (An et al., 2003). The neuroadapted D4MB-6 strain used in our laboratory inoculated intraperitoneally or by footpad injection in 3-day-old Balb/c mice produced encephalitis and paralysis.

Once the virus reaches the brain, DENV induces paralysis, myelitis, and transverse myelitis in addition to unconsciousness and behavioral changes, indicating the neuronal involvement during infection. Neuronal apoptosis appears to be responsible for neurological manifestations, at least in infected mice, as hippocampal and cortical neurons die during infection, many of them undergoing apoptotic changes (Deprès et al., 1996). In this regard, the neuroadapted D4MB-6 strain induced a high proportion of TUNEL positive cells in the brain parenchyma (Velandia-Romero et al., 2012), similar to the findings in adult C57/BL6 mice which were intracerebrally inoculated with DENV3. They suffered behavioral changes associated with hippocampal neuron apoptosis mediated by caspase-3 (Silva de Miranda et al., 2012), presumably related to high levels of TNF-α secretion by neurons or glia (Jhan et al., 2018). It is not clear if at least part of the death seen in neurons is due to a bystander effect rather than direct infection.

Neuronal death and apoptosis have been frequently reported in cultures, showing a pattern of chromatin condensation by electronic microscopy and DNA fragmentation (TUNEL positive) (Ludlow et al., 2016). This finding also occurred in N18 mouse neuroblastoma cells, which showed additional arrest in the G1 cell cycle phase (Su et al., 2001). Recently Ho et al. (2017), described cell death, proliferation arrest, and morphological changes in Neuro-2a cells after DENV2 infection using a high MOI (Ho et al., 2017). Although these results are obtained in mouse neuroblastoma cells, we recently reported that DENV2 infection in SH-SY5Y human neuroblastoma cells induces cell death, expression, and secretion of TNF-α as well as DNA fragmentation and Annexin V translocation, suggesting similar responses among species and validating these models (Castellanos J. E. et al., 2016).

## Conclusion

Dengue pathogenesis is complex and sometimes may appear as nervous system damage, with neurological signs and symptoms. Clinical evidence, as well as evidence in cells and animals, suggests that DENV may enter the CNS via the hematogenous route and infect both neurons and microglial cells, before eventually infecting the EC of BBB. These infected cells, when activated, prevent virus spread and help to control their deleterious effects. This response appears to be adequate in most human cases and controls infection and inflammation with minor or no sequelae; however, some cases result in severe disease or death. The studies in cells and mice show apoptotic neuronal death associated with direct infection due to inflammatory mediators or excitotoxicity. Glial cell and EC responses during infection indicate that soluble molecules, cytokines, and chemokines such as RANTES, TNF-α, IL-6, IL-10, and MCP-1 among others, favor leukocyte infiltration, worsening brain inflammation. This response is standard for a neurotropic *Flavivirus* such as JEV and WNV and indicates a putative neurotropic feature of dengue. Immune responses in the nervous system may also be responsible for the loss of barrier function, allowing a massive infiltration of infected/activated monocytes that enhance viral spread and tissue injury, establishing a vicious circle of high permeability, greater infiltration, greater cell infection, and greater inflammation.

## Author Contributions

M-AC-P formulated the review idea, and wrote, translated, and edited the review. MV-R formulated the review idea, and wrote and edited the review. LB-L, EB, and SC-O wrote and edited the review. JC wrote, translated, and edited the review.

## Conflict of Interest Statement

The authors declare that the research was conducted in the absence of any commercial or financial relationships that could be construed as a potential conflict of interest.

## References

[B1] Abbott N. Rönnbäck L. Hans E. (2006). Astrocyte–endothelial interactions at the blood–brain barrier. *Nat. Rev. Neurosci.* 7 41–53. 10.1038/nm1824 16371949

[B2] Abounader R. Villemure J. Hamel E. (1995). Characterization of neuropeptide Y (NPY) receptors in human cerebral arteries with selective agonists and the new Y1 antagonist BIBP 3226. *Br. J. Pharmacol.* 116 2245–2250. 10.1111/j.1476-5381.1995.tb15060.x 8564255 PMC1908978

[B3] Abraham S. Manjunath R. (2006). Induction of classical and nonclassical MHC-I on mouse brain astrocytes by Japanese encephalitis virus. *Virus Res.* 119 216–220. 10.1016/j.virusres.2006.03.006 16621104

[B4] Abraham S. Yaddanapudi K. Thomas S. Damodaran A. Ramireddy B. Manjunath R. (2008). Nonclassical MHC-I and Japanese encephalitis virus infection: Induction of H-2Q4. H-2T23 and H-2T10. *Virus Res.* 133 239–249. 10.1016/j.virusres.2007.12.023 18314211

[B5] Al-Fifi Y. Kadkhoda K. Drebot M. Wudel B. Bow E. (2018). The first case report of west nile virus-induced acute flaccid quadriplegia in Canada. *Case Rep. Infect Dis.* 2018:4361706 30123588 10.1155/2018/4361706 PMC6079605

[B6] An J. Zhou D.-S. Kawasak K. Yasui K. (2003). The pathogenesis of spinal cord involvement in dengue virus infection. *Virchows Arch.* 442 472–481. 10.1007/s00428-003-0783-783 12695911

[B7] Andrews B. S. Theofilopoulos A. N. Peters C. J. Loskutoff D. J. Brandt W. E. Dixon F. J. (1978). Replication of dengue and junin viruses in cultured rabbit and human endothelial cells. *Infect. Immun.* 20 776–781. 208980 10.1128/iai.20.3.776-781.1978 PMC421926

[B8] Angibaud G. Luaute J. Laille M. Gaultier C. (2001). Brain involvement in dengue fever. *J. Clin. Neurosci.* 8 63–65. 10.1054/jocn.2000.0735 11148085

[B9] Arunachalam N. Tana S. Espino F. Kittayapong P. Abeyewickreme W. Wai K. (2010). Eco-bio-social determinants of dengue vector breeding: a multicountry study in urban and periurban Asia. *Bull. World Health Organ.* 88 173–184. 10.2471/BLT.09.067892 20428384 PMC2828788

[B10] Asnis D. S. Conetta R. Teixeira A. A. Waldman G. Sampson B. A. (2000). The West Nile virus outbreak of 1999 in New York: the flushing hospital experience. *Clin. Infect. Dis.* 30 413–418. 10.1086/313737 10722421

[B11] Aspelund A. Antila S. Proulx S. Karlsen T. Karaman S. Detmar M. (2015). A dural lymphatic vascular system that drains brain intersritial fluid and macromolecules. *J. Exp. Med.* 212:991.99. 10.1084/jem.20142290 26077718 PMC4493418

[B12] Avirutnan P. Malasit P. Seliger B. Sucharit B. Hussmann M. (1998). Cells leads to chemokine production. dengue virus infection of human endothelial complement activation, and apoptosis. *J. Immunol.* 161 6338–6346. 9834124

[B13] Avirutnan P. Punyadee N. Noisakran S. Komoltri C. Thiemmeca S. Auethavornanan K. (2006). Vascular leakage in severe dengue virus infections: a potential role for the nonstructural viral protein NS1 and complement. *J. Infect. Dis.* 193 1078–1088. 10.1086/500949 16544248

[B14] Azmin S. Sahathevan R. Suehazlyn Z. Rabani R. Nafisah W. Tan H. (2013). Post-dengue parkinsonism. *BMC Infect. Dis.* 13:179. 10.1186/1471-2334-13-179 23594500 PMC3637512

[B15] Basu A. Chaturvedi U. C. (2008). Vascular endothelium: the battlefield of dengue viruses. *FEMS Immunol. Med. Microbiol.* 53 287–299. 10.1111/j.1574-695X.2008.00420.x 18522648 PMC7110366

[B16] Baymakova M. Trifonova I. Panayotova E. Dakova S. Pacenti M. Barzon L. (2016). Fatal case of west nile neuroinvasive disease in bulgaria. *Emerg. Infect. Dis.* 22 2203–2204. 10.3201/eid2212.151968 27392134 PMC5189127

[B17] Bhatt R. Kothari S. Gohil D. D’Souza M. Chowdhary A. (2015). Novel evidence of microglial immune response in impairment of dengue infection of CNS. *Immunobiology* 220 1170–1176. 10.1016/j.imbio.2015.06.002 26074064

[B18] Bhatt S. Gething P. Brady O. Messina J. Farlow A. Moyes C. (2013). The global distribution and burden of dengue. *Nature* 496 504–507. 10.1038/nature12060 23563266 PMC3651993

[B19] Bhoopat L. Bhamarapravati N. Attasiri C. Yoksarnl S. Chaiwun B. Khunamornpong S. (1996). Immunohistochemical characterization of a new monoclonal antibody reactive with dengue virus-infected cells in frozen tissue using immunoperoxidase technique. *Asian Pac. J. Allergy Immunol.* 14 107–113. 9177824

[B20] Bhowmick S. Duseja R. Das S. Appaiahgiri M. B. Vrati S. Basu A. (2007). Induction of IP-10 (CXCL10) in astrocytes following Japanese encephalitis. *Neurosci. Lett.* 414 45–50. 10.1016/j.neulet.2006.11070 17287085

[B21] Boonnak K. Dambach K. Donofrio G. Tassaneetrit B. Marovich M. (2011). Cell type specificity and host genetic polymorphisms influence antibody-dependent enhancement of dengue virus infection. *J. Virol.* 85 1671–1683. 10.1128/JVI.00220-210 21123382 PMC3028884

[B22] Bopeththa B. Ralapanawa U. (2017). Post encephalitic parkinsonism following dengue viral infection. *BMC Res. Notes* 10:655. 10.1186/s13104-017-2954-2955 29187231 PMC5708097

[B23] Buffo A. Rolando C. Ceruti S. (2010). Astrocytes in the damaged brain: molecular and cellular insights into their reactive response and healing potential. *Biochem. Pharmacol.* 79 77–89. 10.1016/j.bcp.2009.09.014 19765548

[B24] Burkert K. Moodley K. Angel C. Brooks A. Graham E. Brooks A. (2012). Detailed analysis of inflammatory and neuromodulatory cytokine secretion from human NT2 astrocytes using multiplex bead array. *Neurochem. Int.* 60 573–580. 10.1016/j.neuint.2011.09.002 21939706

[B25] Calvert J. K. Helbig K. J. Dimasi D. Cockshell M. Beard M. R. Pitson S. M. (2015). Dengue virus infection of primary endothelial cells induces innate immune responses. changes in endothelial cells function and is restricted by interferon-stimulated responses. *J. Interferon. Cytokine Res.* 35 654–65. 10.1089/jir.2014.0195 25902155

[B26] Cardier J. Rivas B. Romano E. Rothman A. Perez-Perez C. Ochoa M. (2006). Evidence of vascular damage in dengue disease: demonstration of high levels of soluble cell adhesion molecules and circulating endothelial cells. *Endothelium.* 13 335–340. 10.1080/10623320600972135 17090406

[B27] Cardoso F. Brites D. Brito M. (2010). Looking at the blood–brain barrier: Molecular anatomy and possible investigation approaches. *Brain Res. Rev.* 64 328–363. 10.1016/j.brainresrev.2010.05.003 20685221

[B28] Castellanos J. E. Neissa J. I. Camacho S. J. (2016). Dengue virus induces apoptosis in SH-SY5Y human neuroblastoma cells. *Biomédica* 36 156–168. 10.7705/biomedical.v36i0.2 27622805

[B29] Castellanos J. Coronel C. Parra S. Castilla M. Calvo E. Arévalo M. (2016). Description of high rates of inapparent and simultaneous multiple dengue virus infection in a Colombian jungle. *Tropical. Biomedicine.* 33 375–382. 33579106

[B30] Cauli B. Tong X. Rancillac A. Serluca N. Lambolez B. Rossier J. (2004). Cortical GABA interneurons in neurovascular coupling: relays for subcortical vasoactive pathways. *J. Neurosci.* 24 8940–8949. 10.1523/JNEUROSCI.3065-04.2004 15483113 PMC6730057

[B31] Cdc. (2011). Japanese encephalitis in two children–United States, 2010. *MMWR Morb. Mortal. Wkly. Rep.* 60 276–278. 21389931

[B32] Chang C. Li J. Chen W. Ou Y. Lai C. Hu Y. (2015). Disruption of in vitro endothelial barrier integrity by Japanese encephalitis virus-Infected astrocytes. *Glia.* 63 1915–1932. 10.1002/glia.22857 25959931

[B33] Chang C. Li J. Ou Y. Lin S. Wang Y. Chen W. (2017). Interplay of inflammatory gene expression in pericytes following Japanese encephalitis virus infections. *Brain Behav. Immun.* 66 230–243. 10.1016/j.bbi.2017.07.003 28690034

[B34] Chantick C. Kanlaya R. Kiatbumrung R. Pattanakisakul S. Thongboonkerd V. (2016). Caveolae-mediated albumin trancytosis is enhanced in dengue-infected human endotelial cells: A model of vascular leakege in dengue hemorrahagic fever. *Sci. Rep.* 6:31855. 10.1038/srep31855 27546060 PMC4992822

[B35] Chaturvedi U. C. Dhawan R. Madhu K. Mathur A. (1991). Breakdown of the blood-brain barrier during dengue virus infection of mice. *J. Gen. Virol.* 72 859–866. 10.1099/0022-1317-72-4-859 1707947

[B36] Chaturvedi U. C. Mathur A. Chandra A. Das S. K. Tandon H. O. Singh U. K. (1980). Transplacental Infection with Japanese Encephalitis Virus. *J. Infect. Dis.* 141 712–715. 10.1093/infdis/141.6.712 6248601

[B37] Cheeran M. Hu S. Sheng W. Rashid A. Peterson P. (2005). Differential responses of human brain cells to West Nile virus infection. *J. Neurovirol.* 11 512–524. 10.1080/13550280500384982 16338745

[B38] Chen C. J. Chen J. H. Chen S. Y. Liao S. L. Raung S. L. (2004). Upregulation of RANTES gene expression in neuroglia by japanese encephalitis virus infection. *J. Gen. Virol.* 78 12107–12119. 10.1128/JVI.78.22.12107-12119.2004 15507597 PMC525064

[B39] Chen C. J. Ou Y. C. Chang C. Y. Pan H. C. Liao S. L. Raung S. L. (2011a). TNF-α and IL-1β mediate Japanese encephalitis virus-induced RANTES gene expression in astrocytes. *Neurochem. Int.* 58 234–242. 10.1016/j.neuint.2010.12.009 21167894

[B40] Chen C. J. Ou Y. C. Chang C. Y. Pan H. C. Lin S. Y. Liao S. L. (2011b). Src signaling involvement in Japanese encephalitis virus-induced cytokine production in microglia. *Neurochem. Int.* 58 924–933. 10.1016/j.neuint.2011.02.022 21354239

[B41] Chen C. J. Ou Y. C. Lin S. Y. Raung S. L. Liao S. L. Lai C. Y. (2010). Glial activation involvement in neuronal death by Japanese encephalitis virus infection. *J. Gen. Virol.* 91 1028–1037. 10.1099/vir.0.013565-13560 20007359

[B42] Chen C. Liao S. Kuo M. Wang Y. (2000). Astrocytic alteration induced by Japanese encephalitis virus infection. *Neuroreport* 11 1933–1937. 10.1097/00001756-200006260-00025 10884046

[B43] Chen C. Ou Y. Li J. Chang C. Pan H. Lai C. (2014). Infection of pericytes in vitro by Japanese Encephalitis Virus disrupts the integrity of the endotelial barrier. *J. Virol.* 88 1150–1161. 10.1128/JVI.02738-2713 24198423 PMC3911661

[B44] Cheng V. C. Sridhar S. Wong S. C. Wong S. C. Chan J. F. Yip C. C. (2018). Japanese Encephalitis Virus transmitted via blood transfusion, Hong Kong, China. *Emerg. Infect. Dis.* 24 49–57. 10.3201/eid2401.171297 29043965 PMC5749455

[B45] Chotmongkol V. Sawanyawisuth K. (2004). Case report: dengue hemorrhagic fever with encephalopathy in an adult. *Southeast Asian J. Trop. Med. Public Health.* 35 160–161. 15272761

[B46] Chretien J. Anyamba A. Small J. Britch S. Sánchez J. Halbach A. (2015). Global climate anomalies and potential infectious disease risks: 2014-2015. *PloS PLoS Curr.* 26:7. 10.1371/currents.outbreaks.95fbc4a8fb4695e049baabfc2fc8289f 25685635 PMC4323421

[B47] Chung C. Lee S. Chen Y. Tsai H. Wann S. Kao C. (2007). Acute flaccid paralysis as an unusual presenting symptom of Japanese encephalitis: a case report and review of the literature. *Infection* 35 30–32. 10.1007/s15010-007-6038-6037 17297587

[B48] Cologna R. Armstrong P. Rico-Hesse R. (2005). Selection for virulent dengue viruses occurs in humans and mosquitoes. *J. Virol.* 79 853–859. 10.1128/JVI.79.2.853-859.2005 15613313 PMC538581

[B49] Cruz-Oliveira C. Freire J. Conceição T. Higa L. Castanho M. Da Poian A. (2015). Receptors and routes of dengue virus entry into the host cells. *FEMS Microbiol. Rev.* 39 155–170. 10.1093/femsre/fuu004 25725010

[B50] Cushing M. M. Brat D. J. Mosunjac M. I. Hennigar R. A. Jernigan D. B. Lanciotti R. (2004). Fatal West Nile Virus Encephalitis in a renal transplant recipient. *Am. J. Clin. Pathol.* 121 26–31. 10.1309/G23C-P54D-AR1B-CT8L 14750237

[B51] Dalrymple N. A. Mackow E. R. (2014). Virus interactions with endothelial cell receptors: implications for viral pathogenesis. *Curr. Opin. Virol.* 7 134–140. 10.1016/j.coviro.2014.06.006 25063986 PMC4206553

[B52] Dalrymple N. Mackow E. R. (2011). Productive dengue virus infection of human endothelial cells is directed by heparan sulfate-containing proteoglycan receptors. *J. Virol.* 85 9478–9485. 10.1128/JVI.05008-11 21734047 PMC3165770

[B53] Das S. Mishra M. K. Ghosh J. Basu A. (2008). Japanese Encephalitis Virus infection induces IL-18 and IL-1β in microglia and astrocytes: correlation with in vitro cytokine responsiveness of glial cells and subsequent neuronal death. *J. Neuroimmunol.* 195 60–72. 10.1016/j.jneuroim.2008.01.009 18374991

[B54] Delcambre G. Liu J. Streit W. Shaw G. Vallario K. Herrington J. (2017). Phenotypic characterization of cell populations in the brains of horses experimentally infected with West Nile virus. *Equine Vet. J.* 49 815–820. 10.1111/evj.12697 28470955

[B55] Deprès P. Flamand M. Ceccaldi P. E. Deubel V. (1996). Human isolates of dengue type 1 virus induce apoptosis in mouse neuroblastoma cells. *J. Virol.* 70 4090–4096. 8648748 10.1128/jvi.70.6.4090-4096.1996 PMC190291

[B56] DeSalvo D. Roy-Chaudhury P. Peddi R. Merchen T. Konijetti K. Gupta M. (2004). West Nile virus encephalitis in organ transplant recipients: another high-risk group for meningoencephalitis and death. *Transplantation* 77 466–469. 10.1097/01.TP.0000101434.98873.CB 14966429

[B57] Descloux E. Cao-Lormeau V. Roche C. De Lamballerie X. (2009). Dengue 1 diversity and microevolution. French Polynesia 2001–2006: connection with epidemiology and clinics. *PLoS Negl. Trop. Dis.* 3:e493. 10.1371/journal.pntd.0000493 19652703 PMC2714178

[B58] Diniz J. A. Da Rosa A. P. Guzman H. Xu F. Xiao S. Y. Popov V. L. (2006). West Nile virus infection of primary mouse neuronal and neuroglial cells: the role of astrocytes in chronic infection. *Am. J. Trop. Med. Hyg.* 75 691–696. 10.4269/ajtmh.2006.75.691 17038696

[B59] Dirlikov E. Torres J. Martines R. Reagan-Steiner S. Pérez G. Rivera A. (2018). Postmortem findings in patient with guillain-barré syndrome and zika virus infection. *Emerg. Infect. Dis.* 24 114–117. 10.3201/eid2401.171331 29261094 PMC5749436

[B60] do Rosário M. de Jesus P. Vasilakis N. Farias D. Novaes M. Rodrigues S. (2016). Guillain-barré syndrome after zika virus infection in Brazil. *Am. J. Trop. Med. Hyg.* 95 1157–1160. 10.4269/ajtmh.16-0306 27645785 PMC5094232

[B61] Doi M. L. Tatsuno S. Singh G. Tatsuno E. Mau M. (2017). Neurological complications in a polynesian traveler with dengue. *Hawaii J. Med. Public Health.* 76 275–278. 29018589 PMC5630466

[B62] Dong Y. Benveniste E. (2001). Immune function of astrocytes. *Glia* 36 180–190. 11596126 10.1002/glia.1107

[B63] Doron S. I. Dashe J. F. Adelman L. S. Brown W. F. Werner B. G. Hadley S. (2003). Histopathologically proven poliomyelitis with quadriplegia and loss of brainstem function due to west nile virus infection. *Clin. Infect. Dis.* 37 e74–e77. 10.1086/377177 12942423

[B64] Doti P. Castro P. Martínez M. J. Zboromyrska Y. Aldasoro E. Inciarte A. (2013). A case of Japanese encephalitis in a 20 year-old spanish sportsman. february 2013. *Euro Surveill.* 18:20573. 10.2807/1560-7917.ES2013.18.35.20573 24008230

[B65] Endy T. Anderson K. Nisalak A. Yoon I. Green S. Rothman A. (2011). Determinants of inapparent and symptomatic dengue infection in a prospective study of primary school children in Kamphaeng Phet Thailand. *PLoS Negl. Trop. Dis.* 5:e975. 10.1371/journal.pntd.0000975 21390158 PMC3046956

[B66] Eng L. Ghirnikar R. Lee Y. (2000). Glial fibrillary acidic protein: GFAP-thirty-one years (1969–2000). *Neurochemistry.* 25 1439–1451. 10.1023/A:1007677003387 11059815

[B67] Fabrizius R. G. Anderson K. Hendel-Paterson B. Kaiser R. M. Maalim S. Walker P. F. (2016). Case report: guillain–barré syndrome associated with zika virus infection in a traveler returning from guyana. *Am. J. Trop. Med. Hyg.* 95 1161–1165. 10.4269/ajtmh.16-0397 27807296 PMC5094233

[B68] Farina C. Aloisi F. Meini E. (2007). Astrocytes are active players in cerebral innate immunity. *Trends Immunol.* 28 138–145. 10.1016/j.it.2007.01.005 17276138

[B69] Fernandes N. Nogueira J. Réssio R. Cirqueira C. Kimura L. Fernandes K. (2017). Experimental Zika virus infection induces spinal cord injury and encephalitis in newborn Swiss mice. *Exp. Toxicol. Pathol.* 69 63–71. 10.1016/j.etp.2016.11.004 27899230

[B70] Fong C. Hlaing C. Tay C. Kadir K. Goh K. Ong L. (2016). Longitudinal extensive transverse myelitis with cervical epidural haematoma following dengue virus infection. *Eur. J. Paediatr. Neurol.* 20 449–453. 10.1016/j.ejpn.2016.01.012 26900103

[B71] Fong C. Khine M. Peter A. Lim W. Rozalli F. (2017). Mild encephalitis/encephalopathy with reversible splenial lesion (MERS) due to dengue virus. *J. Clin. Neurosci.* 36 73–75. 10.1016/j.jocn.2016.10.050 27887978

[B72] Garber C. Vasek M. J. Vollmer L. L. Sun T. Jiang X. Klein R. S. (2018). Astrocytes decrease adult neurogenesis during virus-induced memory dysfunction via interleukin-1. *Nat. Immunol.* 19 151–161. 10.1038/s41590-017-0021-y 29292385 PMC5786497

[B73] Garcia-Ponce A. Citalán M. Velázquez M. Vargas H. Schnoor M. Thromb H. (2015). The role of actin-binding proteins in the control of endothelial barrier integrity. *Thromb. Haemost.* 113 20–36. 10.1160/TH14-04-0298 25183310

[B74] George S. Gourie-Devie M. Rao J. A. Prasad R. Pavri K. (1984). Isolation of West Nile virus from the brains of children who had died of encephalitis. *Bull. World Health Organ.* 62 879–882. 6099760 PMC2536262

[B75] German A. Myint K. Mai N. Pomeroy I. Phu N. Tzartos J. (2006). A preliminary neuropathological study of Japanese encephalitis in humans and a mouse model. *Trans. R Soc. Trop. Med. Hyg.* 100 1135–1145. 10.1016/j.trstmh.2006.02.008 16814333

[B76] Giladi M. Metzkor-Cotter E. Martin D. A. Siegman-Igra Y. Korczyn A. D. Rosso R. (2001). West Nile encephalitis in Israel, 1999: the New York connection. *Emerg. Infect. Dis.* 7 659–661. 10.3201/eid704.010410 11585528 PMC2631756

[B77] Ginhoux F. Prinz M. (2015). Origin of microglía: current concepts and past controversies. *Cold Spring Harb. Perspect. Biol.* 7:a020537. 10.1101/cshperspect.a020537 26134003 PMC4526747

[B78] Goswami R. Mukherjee A. Biswas T. Karmakar P. Ghosh A. (2012). Two cases of dengue meningitis: a rare first presentation. *J. Infect. Dev. Ctries.* 6 208–211. 10.3855/jidc.2241 22337854

[B79] Gotoh J. Kuang T. Nakao Y. Cohen D. Melzer P. Itoh Y. (2001). Regional differences in mechanisms of cerebral circulatory response to neuronal activation. *Am. J. Physiol. Heart Circ. Physiol.* 280 H821–H829. 10.1152/ajpheart.2001.280.2H821 11158982

[B80] Grove J. Marsh M. (2011). The cell biology of receptor-mediated virus entry. *J. Cell Biol.* 195 1071–1082. 10.1083/jcb.201108131 22123832 PMC3246895

[B81] Gulati S. Maheshwari A. (2007). Atipical manifestations of dengue. *Trop. Med. Int. Health* 12 1087–1095. 10.1111/j.1365-3156.2007.01891.x 17875019

[B82] Guzman M. G. Harris E. (2015). Dengue. *Lancet* 385 453–465. 10.1016/S0140-6736(14)60572-60579 25230594

[B83] Guzman M. G. Kouri G. Bravo J. Valdés L. Vázquez S. Halstead S. (2002). Effect of age on outcome of secondary dengue 2 infections. *Int. J. Infect. Dis.* 6 118–124. 10.1016/S1201-9712(02)90072-X 12121599

[B84] Hapuarachchi H. Chua R. Shi Y. Thein T. Lee L. Lee K. (2015). Clinical Outcome and genetic differences within a monophyletic dengue virus type 2 population. *PLoS One.* 10:e0121696. 10.1371/journal.pone.0121696 25811657 PMC4374945

[B85] Hapuarachchi H. Oh H. Thein T. Pok K. Lai Y. Tan L. (2013). Clinico-genetic characterisation of an encephalitic Dengue virus 4 associated with multi-organ involvement. *J. Clin. Virol.* 57 91–94. 10.1016/j.jcv.2012.12.021 23415634

[B86] Hawkes M. Toledano M. Kaufmann T. Rabinstein A. (2018). West Nile neuroinvasive disease presenting as elsberg syndrome. *Neurologist.* 23 152–154. 10.1097/NRL.0000000000000189 30169366

[B87] Ho M. R. Tsai T. T. Chen C. L. Jhan M. K. Tsai C. C. Lee Y. C. (2017). Blockade of dengue virus infection and viral cytotoxicity in neuronal cells in vitro and in vivo by targeting endocytic pathways. *Sci. Rep.* 7:6910. 10.1038/s41598-017-07023-z 28761128 PMC5537343

[B88] Hotta H. Murakami I. Miysaki K. Takeda Y. Shirane H. Hotta S. (1981). Localization of Dengue Virus in nude mice. *Microbiol. Immunol.* 25 89–93. 10.1111/j.1348-0421.1981.tb00012.x 7253962

[B89] Hou J. Baker L. A. Zhou L. Klein R. S. (2016). Viral interactions with the blood-brain barrier: old dog, new tricks. *Tissue Barriers.* 4:e1142492. 10.1080/21688370.2016.1142492 27141421 PMC4836465

[B90] Hunsperger E. Roehrig J. (2009). Nocodazole delays viral entry into the brain following footpad inoculation with West Nile virus in mice. *J. Neurovirol.* 15 211–218. 10.1080/13550280902913255 19444694

[B91] Hussmann K. L. Fredericksen B. L. (2014). Differential induction of CCL5 by pathogenic and non-pathogenic strains of West Nile virus in brain endothelial cells and astrocytes. *J. Gen. Virol.* 95(Pt 4), 862–867. 10.1099/vir.0.060558-60550 24413421 PMC3973477

[B92] Hwang J. Ryu H. Kim H. Lee S. (2015). The first reported case of West Nile encephalitis in Korea. *J. Korean Med. Sci.* 30 343–345. 10.3346/jkms.2015.30.3.343 25729260 PMC4330492

[B93] Iliff J. Wang M. Liao Y. Plogg B. Peng W. Gundersen G. (2012). A Paravascular pathway facilitates CSF flow through the brain parenchyma and the clearance of interstitial solutes, including Amyloid β. *Sci. Transl. Med.* 4:147ra111. 10.1126/scitranslmed.3003748 22896675 PMC3551275

[B94] Imbert J. L. Guevara P. Ramos-Castañeda J. (1994). Dengue virus infects mouse cultured neurons but not astrocytes. *J. Med. Virol.* 42 228–233. 10.1002/jmv.1890420304 8006634

[B95] Iwamoto M. Jernigan D. B. Guasch A. Trepka M. J. Blackmore C. G. Hellinger W. (2003). Transmission of West Nile Virus from an organ donor to four transplant recipients. *N. Engl. J. Med.* 348 2196–2203. 10.1056/NEJMoa022987 12773646

[B96] Jessie K. Fong M. Y. Devi S. Lam S. K. Wong K. T. (2004). Localization of dengue virus in naturally infected human tissues, by immunohistochemistry and in situ hybridization. *J. Med. Virol.* 189 1411–1418. 10.1086/383043 15073678

[B97] Jha M. Jo M. Kim J. Suk K. (2018). Microglial-astrocyte crosstalk: an intimate molecular convesrsation. *Neuroscientist.* 25 227–240. 10.1177/1073858418783959 29931997

[B98] Jhan M. K. Huang Fu W. C. Chen Y. F. Kao J. C. Tsai T. T. Ho M. R. (2018). Anti-TNF-α restricts dengue virus-induced neuropathy. *J. Leukoc Biol.* 104 961–968. 10.1002/JLB.MA1217-484R 30044892

[B99] Jhan M. Tsai T. Chen C. Tsai C. Cheng Y. Lee Y. (2017). Dengue virus infection increases microglial cell migration. *Sci. Rep.* 7:91. 10.1038/s41598-017-00182-z 28273893 PMC5427823

[B100] Jia N. Zhao Q. Guo X. Cheng J. Wu C. Zuo S. (2011). Encephalitis temporally associated with live attenuated Japanese encephalitis vaccine: four case reports. *BMC Infect. Dis.* 11:344. 10.1186/1471-2334-11-344 22168358 PMC3282669

[B101] Kelley T. Prayson R. A. Ruiz A. I. Isada C. M. Gordon S. M. (2003). The Neuropathology of West Nile Virus *Meningoencephalitis*. *Am. J. Clin. Pathol.* 119 749–753. 10.1309/PU4R-76JJ-MG1F-81RP 12760295

[B102] Kigerl K. de Rivero Vaccari J. Dietrich W. Popovich P. Keane R. (2014). Pattern recognition receptors and central nervous system repair. *Exp. Neurol.* 258 5–16. 10.1016/j.expneurol.2014.01.001 25017883 PMC4974939

[B103] Krasowska-Zoladek A. Banaszewska M. Kraszpulski M. Konat G. (2007). Kinetics of inflammatory response of astrocytes induced by TLR 3 and TLR4 ligation. *J. Neurosci. Res.* 85 205–212. 10.1002/jnr.21088 17061254

[B104] Kumar M. Verma S. Nerurkar V. (2010). Pro-inflammatory cytokines derived from West Nile virus (WNV)-infected SK-N-SH cells mediate neuroinflammatory markers and neuronal death. *J. Neuroinflammation.* 7 1–14. 10.1186/1742-2094-7-73 21034511 PMC2984415

[B105] Kuwar R. B. Stokic D. S. Leis A. A. Bai F. Paul A. M. Frantkin J. D. (2015). Does astroglial protein S100B contribute to West Nile neuro-invasive syndrome? *J. Neurol. Sci.* 358 243–252. 10.1016/j.jns.2015.09.003 26382833

[B106] Landrith T. A. Harris T. Wilson E. (2015). Characteristics and critical function of CD8+ T cells in the Toxoplasma-infected brain. *Semin. Immunopathol.* 37 261–270. 10.1007/s00281-015-0487-483 25898888 PMC5313077

[B107] Langevin S. Libman M. Drebot M. A. Laverdiere M. (2012). A Case of Japanese Encephalitis Virus Infection Acquired During a Trip in Thailand. *J. Travel Med.* 19 127–129. 10.1111/j.1708-8305.2011.00582 22414040

[B108] Lannes N. Eppler E. Etemad S. Yotovski P. Filgueira L. (2017). Microglia at center stage: a comprehensive review about the versatile and unique residential macrophages of the central nervous system. *Oncotarget* 8 114393–114413. 10.18632/oncotarget.23106 29371994 PMC5768411

[B109] Lanteri M. Busch M. (2012). Dengue in the context of “safe blood” and global epidemiology: to screen or not to screen? *Transfusion* 52 1634–1639. 10.1111/j.1537-2995.2012.03747.x 22882092 PMC3509801

[B110] Lee K. M. Chiu K. B. Sansing H. A. Didier P. J. Lackner A. A. MacLean A. G. (2016). The Flavivirus Dengue induces hypertrophy of white matter astrocytes. *J. Neurovirol.* 22 831–839. 10.1007/s13365-016-0461-464 27273075 PMC5130605

[B111] Lehtinen V. Huhtamo E. Siikamäki H. Vapalahti O. (2008). Japanese encephalitis in a Finnish traveler on a two-week holiday in Thailand. *J. Clin. Virol.* 43 93–95. 10.1016/j.jcv.2008.05.001 18556242

[B112] Lei S. Tian Y. Xiao W. Li S. Rao X. Zhang J. (2013). ROCK is involved in vimentin phosphorylation and rearrangement induced by dengue virus. *Cell Biochem. Biophys.* 67 1333–1342. 10.1007/s12013-013-9665-x 23737341 PMC3838595

[B113] Li G. Ning Z. Liu Y. Li X. (2017). Neurological manifestations of dengue infection. *Front Cell Infect. Microbiol.* 7:449. 10.3389/fcimb.2017.00449 29119088 PMC5660970

[B114] Lima-Juniora R. S. da Silva Mello C. Siani A. C. Valente L. M. Fernandes Kubelk C. (2013). Uncaria tomentosa alkaloidal fraction reduces paracellular permeability. IL-8 and NS1 production on human microvascular endothelial cells infected with dengue virus. *Nat. Prod. Commun.* 8 1547–1550. 24427938

[B115] Limonta D. Capo V. Torres G. Perez A. Guzmán M. G. (2007). Apoptosis in tissues from fatal dengue shock syndrome. *J. Clin. Virol.* 40 50–54. 10.1016/j.jcv.2007.04.024 17693133

[B116] Limonta D. Falcón V. Torres G. Capó V. Menéndez I. Rosario D. (2012). Dengue virus identification by transmission electron microscopy and molecular methods in fatal dengue hemorrhagic fever. *Infection.* 40 689–694. 10.1007/s15010-012-0260-267 22527878

[B117] Lindqvist R. Mundt F. Gilthorpe J. Wölfel S. Gekara N. Kröger A. (2016). Fast type I interferon response protects astrocytes from flavivirus infection and virus-induced cytopathic effects. *J. Neuroinflammation* 13:277. 10.1186/s12974-016-0748-747 27776548 PMC5078952

[B118] Liou M. L. Hsu C. Y. (1998). Japanese encephalitis virus is transported across the cerebral blood vessels by endocytosis in mouse brain. *Cell Tissue Res.* 293 389–394. 9716728 10.1007/s004410051130

[B119] Liu Y. King N. Kesson A. Blanden R. V. Mullbacher A. (1988). West nile virus infection modulates the expression of class I and class I1 mhc antigens on astrocytes in vitro. *Ann. N. Y. Acad. Sci.* 540 483–485. 10.1111/j.1749-6632.1988.tb27143.x 2849899

[B120] Lossia O. Conway M. Tree M. Williams R. Goldthorpe S. Srinageshwar B. (2018). Zika virus induces astrocyte differentiation in neural stem cells. *J. Neurovirol.* 24 52–61. 10.1007/s13365-017-0589-x 29063515

[B121] Louveau A. Harris T. Kipnis J. (2015). Revisiting the concept of CNS immune privilege. *Trends Immunol.* 36 569–577. 10.1016/j.it.2015.08.006 26431936 PMC4593064

[B122] Ludlow M. Kortekaas J. Herden C. Hoffmann B. Tappe D. Trebst C. (2016). Neurotropic virus infections as the cause of immediate and delayed neuropathology. *Acta Neuropathol.* 131 159–184. 10.1007/s00401-015-1511-1513 26659576 PMC4713712

[B123] Lühn K. Simmons C. Moran E. Dung N. Chau T. Quyen N. (2007). Increased frequencies of CD4+ CD25 (high) regulatory T cells in acute dengue infection. *J. Exp. Med.* 204 979–985. 10.1084/jem.20061381 17452519 PMC2118571

[B124] Lum F. Low D. Fan Y. Tan J. Lee B. Chan J. (2017). Zika virus infects human fetal brain microglia and induces inflammation. *Clin. Infect. Dis.* 64 914–920. 10.1093/cid/ciw878 28362944

[B125] Lum L. Lam S. Choy Y. George R. Harun F. (1996). Dengue encephalitis: a true entity? *Am. J. Trop. Med. Hyg.* 54 256–259. 10.4269/ajtmh.1996.54.256 8600761

[B126] Luplertlop N. Missé D. Bray D. Deleuze V. Gonzalez J. Leardkamolkarn V. (2006). Dengue-virus-infected dendritic cells trigger vascular leakage through metalloproteinase overproduction. *EMBO Rep.* 7 1176–1181. 10.1038/sj.embor.7400814 17028575 PMC1679776

[B127] Mancera-Páez O. Román G. Pardo-Turriago R. Rodríguez Y. Anaya J. (2018). Concurrent Guillain-Barré syndrome, transverse myelitis and encephalitis post-Zika: A case report and review of the pathogenic role of multiple arboviral immunity. *J. Neurol. Sci.* 395 47–53. 10.1016/j.jns.2018.09.028 30292020

[B128] Martina B. Koraka P. Osterhaus A. (2009). Dengue virus pathogenesis: an integrated view. *Clin. Microbiol. Rev.* 22 564–581. 10.1128/CMR.00035-39 19822889 PMC2772360

[B129] Mathew R. Basti R. Hegde P. Devdas J. Khan H. Bukelo M. (2014). Rare case of acute dengue encephalitis with correlated MRI findings. *J. Med. Imaging Radiat. Oncol.* 58 679–682. 10.1111/1754-9485.12182 24767167

[B130] Mc Minn P. (1997). The molecular basis of virulence of the encephalitogenic flaviviruses. *J. Gen. Virol.* 78(Pt 11), 2711–2722. 10.1099/0022-1317-78-11-27-11 9367356

[B131] McConnell H. Kersch C. Woltjer R. Neuwelt E. (2017). The translational significance of the neurovascular unit. *J. Biol. Chem.* 292 762–770. 10.1074/jbc.R116.760215 27920202 PMC5247651

[B132] McGavern D. B. Kang S. S. (2011). Illuminating viral infections in the nervous system. *Nat. Rev. Immunol.* 11 318–329. 10.1038/nri2971 21508982 PMC5001841

[B133] Meertens L. Labeau A. Dejarnac O. Cipriani S. Sinigaglia L. Bonnet-Madin L. (2017). Axl mediates ZIKA virus entry in human glial cells and modulates innate immune responses. *Cell Rep.* 18 324–333. 10.1016/j.celrep.2016.12.045 28076778

[B134] Méndez A. González G. (2006). Abnormal clinical manifestations of dengue hemorrhagic fever in children. *Biomédica* 26 61–70. 16929904

[B135] Middeldorp J. Hol E. (2011). GFAP in health and disease. *Prog. Neurobiol.* 93 421–443. 10.1016/j.pneurobio.2011.01.005 21219963

[B136] Miljkovic D. Momcilovic M. Stojanovic I. Stosic-Grujicic S. Ramic Z. Stojkov M. (2007). Astrocytes stimulate interleukin-17 and interferon-gamma production in vitro. *J. Neurosci.* 85 3598–3606. 10.1002/jnr.21453 17969033

[B137] Miller S. Kastner S. Krijnse-Locker J. Bühler S. Bartenschlager R. (2007). The non-structural protein 4A of dengue virus is an integral membrane protein inducing membrane alterations in a 2K-regulated manner. *J. Biol. Chem.* 282 8873–8882. 10.1074/jbc.m609919200 17276984

[B138] Miner J. Diamond M. (2016). Mechanisms of restriction of viral neuroinvasion at the blood-brain barrier. *Curr. Opin. Immnunol.* 38 18–23. 10.1016/j.coi.2015.10.008 26590675 PMC4715944

[B139] Mishra M. Koli P. Bhowmick S. Basu A. (2007). Neuroprotection conferred by astrocytes is insufficient to protect animals from succumbing to Japanese encephalitis. *Neurochem. Int.* 50 764–773. 10.1016/j.neuint.2007.01.2014 17353066

[B140] Mishra M. Kumawat K. Basu A. (2008). Japanese encephalitis virus differentially modulates the induction of multiple pro-inflammatory mediators in human astrocytoma and astroglioma cell-lines. *Cell Biol. Int.* 32 1506–1513. 10.1016/j.cellbi.2008.08.020 18801452

[B141] Mittal M. Jain N. (2011). Subdural haematoma and axonal polyneuropathy complicating dengue fever. *BMJ Case Rep.* 2011:bcr1220103672. 10.1136/bcr.12.2010.3672 22692494 PMC3118898

[B142] Mohta S. Ray A. Sharma S. Vyas S. (2017). Longitudinally extensive transverse myelitis (LETM) in a case of Japanese encephalitis with an unexpected complication. *J. Vector Borne Dis.* 54 291–293. 10.4103/0972-9062.217623 29097647

[B143] Molko N. Simon O. Guyon D. Biron A. Dupont-Rouzeyro M. Gourinat A. C. (2017). Zika virus infection and myasthenia. *Neurology* 88 1097–1098. 10.1212/WNL.0000000000003697 28188305

[B144] Monel B. Compton A. Bruel T. Amraoui S. Burlaud-Gaillard J. Roy N. (2017). Zika virus induces massive cytoplasmic vacuolization and paraptosis-like death in infected cells. *EMBO J.* 36 1653–1668. 10.15252/embj.201695597 28473450 PMC5470047

[B145] Mota M. T. Estofolete C. Zini N. Terzian A. Gongora D. Maia I. (2017). Transverse myelitis as an unusual complication of dengue fever. *Am. J. Trop. Med. Hyg.* 96 380–381. 10.4269/ajtmh.16-0284Bopeththa 27956656 PMC5303040

[B146] Murthy J. (2010). Neurological complications of dengue infections. *Neurol. India.* 58 581–584. 10.4103/0028-3886.68654 20739796

[B147] Myint K. Kipar A. Jarman R. Gibbons R. Perng G. Flanagan B. (2014). Neuropathogenesis of Japanese encephalitis in a primate model. *PLoS Negl. Trop. Dis.* 8:e2980. 10.1371/journal.pntd.0002980 25102067 PMC4125110

[B148] Nadarajah J. Madhusudhan K. Yadav A. Gupta A. Vikram N. (2015). Acute hemorrhagic encephalitis: An unusual presentation of dengue viral infection. *Indian J. Radiol. Imaging.* 25 52–55. 10.4103/0971-3026.150145 25709166 PMC4329688

[B149] Nain M. Abdin M. Kalia M. Vrati S. (2016). Japanese encephalitis virus invasión of cell: allies and alleys. *Rev. Med. Virol.* 26 129–141. 10.1002/rmv.1868 26695690

[B150] Narayanan S. Thulaseedharan N. Subramaniam G. Panarkandi G. Shameer V. Narayanan A. (2017). Hypothermia due to limbic system involvement and longitudinal myelitis in a case of Japanese encephalitis: a case report from India. *Int. J. Gen. Med.* 10 125–129. 10.2147/IJGM.S129829 28442926 PMC5396945

[B151] Nascimento O. Frontera J. Amitrano D. Bispo de Filippis A. Da Silva I. Group R.-G.-Z. R. (2017). Zika virus infection-associated acute transient polyneuritis. *Neurology* 88 2330–2332. 10.1212/WNL.0000000000004026 28500225

[B152] Ng D. H. Sadarangani S. P. (2017). Locked in a cage”. a case of dengue virus 4 encephalitis. *PLoS Negl. Trop. Dis.* 11:e0005369. 10.1371/journal.pntd.0005369 28472166 PMC5417410

[B153] Nogueira R. Filippis A. Sequeira P. Paiva F. Ramos A. Miagostovich M. (2002). Dengue virus infection of the central nervous system (CNS): A case report from Brazil. *Southeast Asian J. Trop. Med. Public Health.* 33 68–71. 12118463

[B154] Obermeier B. Daneman R. Ransohoff R. M. (2013). Development, maintenance and disruption of the blood-brain barrier. *Nat. Med.* 19 1584–1596. 10.1038/nm.3407 24309662 PMC4080800

[B155] Ohainle M. Balmaseda A. Macalalad A. Tellez Y. Zody M. Saborio S. (2011). Dynamics of dengue disease severity determined by the interplay between viral genetics and serotype-specific immunity. *Sci. Transl. Med.* 3:114ra28. 10.1126/scitranslmed.3003084 22190239 PMC4517192

[B156] Patabendige A. Michael B. Craig A. Solomon A. (2018). Brain microvascular endothelial-astrocyte cell responses following Japanese encephalitis virus infection in an in vitro human blood-brain barrier model. *Mol. Cell. Neurosci.* 89 60–70. 10.1016/j.mcn.2018.04.002 29635016 PMC5984247

[B157] Peng H. Liu B. Yves T. He Y. Wang S. Tang H. (2018). Zika Virus Induces Autophagy in Human Umbilical Vein Endothelial Cells. *Viruses.* 10:E259. 10.3390/v10050259 29762492 PMC5977252

[B158] Perea G. Navarrete M. Araque A. (2009). Tripartite synapses: astrocytes process and control synaptic information. *Trends Neurosci.* 32 421–431. 10.1016/j.tins.2009.05.001 19615761

[B159] Peyrefitte C. N. Pastorino B. Grau G. E. Lou J. Tolou H. Couissinier-Paris P. (2006). Dengue virus infection of human microvascular endothelial cells from different vascular beds promotes both common and specific functional changes. *J. Med. Virol.* 78 229–242. 10.1002/jmv.20532 16372301

[B160] Pierson T. Diamond M. (2013). “Flavivirus,” in *Fields Virology*, 6th Edn, eds David M. Peter K. Howley M. Jeffrey I. (Philadelphia: Wolters Kluwer Health/Lippincott Williams & Wilkins), 747–794.

[B161] Plummer E. Shresta S. (2014). Mouse models for dengue vaccines and antivirals. *J Immunol. Meth.* 410 34–38. 10.1016/j.jim.2014.01.001 24440090

[B162] Póvoa T. Alves A. Oliveira C. Nuovo G. Chagas V. Paes M. (2014). The pathology of severe dengue in multiple organs of human fatal cases: histopathology, ultrastructure and virus replication. *PLoS One* 9:e83386. 10.1371/journal.pone.0083386 24736395 PMC3987999

[B163] Pradhan F. Burns J. Agameya A. Patel A. Alfaqih M. Small J. (2017). Case report: zika virus meningoencephalitis and myelitis and associated magnetic resonance imaging findings. *Am. J. Trop. Med. Hyg.* 97 340–343. 10.4269/ajtmh.16-0921 28829736 PMC5544082

[B164] Puccioni-Sohler M. Rosadas C. (2015). Advances and new insights in the neuropathogenesis of dengue infection. *Arq. Neuropsiquiatr.* 73 698–703. 10.1590/0004-282X20150074 26222363

[B165] Puccioni-Sohler M. Orsini M. Soares C. (2012). Dengue: a new challenge for neurology. *Neurol. Int.* 4 e15. 10.4081/ni.2012.e15 23355928 PMC3555217

[B166] Puerta-Guardo H. Glasner D. Harris E. (2016). Dengue virus NS1 disrupts the endothelial glycocalyx. leading to hyperpermeability. *PLoS Pathog.* 12:e1005738. 10.1371/journal.ppat.1005738 27416066 PMC4944995

[B167] Puerta-Guardo H. Raya-Sandino A. González-Mariscal L. Rosales V. Ayala-Dávila J. Chavez-Mungía B. (2013). The cytokine response of U937-derived macrophages infected through antibody-dependent enhancement of dengue virus disrupts cell apical-junction complexes and increases vascular permeability. *J. Virol.* 87 7486–7501. 10.1128/JVI.00085-13 23616663 PMC3700291

[B168] Quick E. D. Smith Leser J. Clarke P. Tyler K. L. (2014). Activation of intrinsic immune responses and microglial phagocytosis in an ex vivo spinal cord slice culture model of west nile virus. *J. Virol.* 88 13005–13014. 10.1128/JVI.01994-1914 25165111 PMC4249089

[B169] Ramesh G. MacLean A. Philipp M. (2013). Cytokines and chemokines at the crossroads of neuroinflammation. neurodegeneration, and neuropathic Pain. *Mediat. Inflammation* 2013:480739. 10.1155/2013/480739 23997430 PMC3753746

[B170] Ramos C. Sánchez G. Hernández R. Baquera J. Hernández D. Mota J. (1998). Dengue virus in the brain of a fatal case of hemorrhagic dengue fever. *J. Neurovirol.* 4 465–468. 9718141 10.3109/13550289809114548

[B171] Ramos-Castañeda J. Imbert J. L. Barron B. L. Ramos C. (1997). A 65-kDa trypsin-sensible membrane cell protein as a possible receptor for dengue virus in cultured neuroblastoma cells. *J. Neuroviol.* 3 435–440. 10.3109/13550289709031189 9475115

[B172] Rao S. Kumar M. Ghosh S. Gadpayle A. (2013). A rare case of dengue encephalitis. *BMJ Case Rep.* 2013:bcr2012008229. 10.1136/bcr-2012-008229 23413293 PMC3604181

[B173] Reddy P. Davenport R. Ratanatharathorn V. Reynolds C. Silver S. Ayash L. (2004). West Nile virus encephalitis causing fatal CNS toxicity after hematopoietic stem cell transplantation. *Bone Marrow Transplant.* 33 109–112. 10.1038/sj.bmt.1704293 14566328

[B174] Retallack H. Di Lullo E. Arias C. Knopp K. Laurie M. Sandoval-Espinosa C. (2016). Zika virus cell tropism in the developing human brain and inhibition by azithromycin. *Proc. Natl. Acad. Sci. U.S.A.* 113 14408–14413. 10.1073/nas1618029113. 27911847 PMC5167169

[B175] Roach T. Alcendor D. (2017). Zika virus infection of cellular components of the blood-retinal barriers implications for viral associated congenital ocular disease. *J. Neuroimmflammation* 14:43. 10.1186/s12974-017-0824-827 28253931 PMC5335843

[B176] Ruhrberg C. Bautch V. (2013). Neurovascular development and links to disease. *Cell. Mol. Life Sci.* 70 1675–1684. 10.1007/s00018-013-1277-1275 23475065 PMC3632722

[B177] Salazar M. I. Pérez-García M. Terreros-Tinoco M. Castro-Mussot M. E. Pérez-Ramirez J. D. Ramírez-Reyes A. G. (2013). Dengue virus type 2: protein binding and active replication in human central nervous system cells. *Scientific. World Journal.* 2013:904067. 10.1155/2013/904067 24302878 PMC3835358

[B178] Salinas S. Schiavo G. Kremer E. J. (2010). A hitchhiker’s guide to the nervous system: The complex journey of viruses and toxins. *Nat. Rev. Microbiol.* 8 645–655. 10.1038/nrmicro2395 20706281

[B179] Sampson B. Armbrustmacher V. (2001). West Nile encephalitis: the neuropathology of four fatalities. *Ann. N. Y. Acad. Sci.* 951 172–178. 10.1111/j.1749-6632.2001.tb02695.x 11797775

[B180] Sandrone S. Moreno Zambrano D. Kipnis J. van Gijn J. (2019). A (delayed) history of the brain lymphatic system. *Nature. Med.* 25 538–540. 10.1038/s41591-019-0417-3 30948855

[B181] Sanguansermsri T. Poneorasert B. Phornphutkul B. (1976). Acute encephalopathy associated with dengue infections. *Bangkok. Seameo Tropmed.* 10–11.

[B182] Schwartzmann P. Ramalho L. Neder L. Vilar F. Ayub-Ferreira S. Romeiro M. (2017). Zika virus meningoencephalitis in an immunocompromised patient. *Mayo Clin. Proc.* 92 460–466. 10.1016/j.mayocp.2016.12.019 28259231

[B183] Scremin O. Rovere A. Raynald A. Giardini A. (1973). Cholinergic control of blood flow in the cerebral cortex of the rat. *Stroke.* 4 233–239. 4702311

[B184] Shaha S. Fite L. P. Lane N. Parekh P. (2016). Purpura fulminans associated with acute West Nile virus encephalitis. *J. Clin. Virol.* 75 1–4. 10.1016/j.jcv.2015.11.034 26686320

[B185] Shao Q. Herrlinger S. Zhu Y. Yang M. Goodfellow F. Stice S. (2017). The African Zika virus MR-766 is more virulent and causes more severe brain damage than current Asian lineage and dengue virus. *Development.* 144 4114–4124. 10.1242/dev.156752 28993398 PMC5719247

[B186] Shweta S. Mahajan R. Jain A. Gadpayle A. (2014). A case of dengue haemorrhagic fever with fatal encephalopathy. *Int. J. Curr. Microbiol. App. Sci.* 3 404–406.

[B187] Sierra B. Kourí G. Guzmán M. G. (2007). Race: a risk factor for dengue hemorrhagic fever. *Arch. Virol.* 152 533–542. 10.1007/s00705-006-0869-x 17106622

[B188] Silva de Miranda A. Rodrigues D. H. Guerra Amaral D. C. de Lima Campos R. D. Cisalpino D. Carvalho Vilela M. (2012). Dengue-3 encephalitis promotes anxiety-like behavior in mice. *Behav Brain Res.* 230 237–242. 10.1016/j.bbr.2012.02.020 22366269

[B189] Simanjuntak Y. Liang J. J. Lin Y. L. (2014). Repurposing of prochlorperazine for use against dengue virus infection. *J. Infect. Dis.* 211 394–404. 10.1093/infdis/jiu377 25028694

[B190] Simonin Y. Loustalot F. Desmetz C. Foulongne V. Constant O. Fournier-Wirth C. (2016). Zika virus strains potentially display different infectious profiles in human neural cells. *EBioMedicine* 12 161–169. 10.1016/j.ebiom.2016.09.020 27688094 PMC5078617

[B191] Siu R. Bukhari W. Todd A. Gunn W. Huang Q. Timmings P. (2016). Acute ZIKA infection with concurrent onset of Guillain-Barré syndrome. *Neurology* 87 1623–1624. 10.1212/WNL.0000000000003038 27466468

[B192] Soe H. J. Khan M. A. Manikam R. Raju C. S. Vanhoutte P. Sekaran S. D. (2017). High dengue virus load differentially modulates human microvascular endothelial barrier function during early infection. *J. Gen. Virol.* 98 2993–3007. 10.1099/jgv.0.000981 29182510

[B193] Sofroniew M. (2009). Molecular dissection of reactive astrogliosis and glial scar formation. *Trends Neurosci.* 32 638–647. 10.1016/j.tins.2009.08.002 19782411 PMC2787735

[B194] Solomão N. Rabelo K. Póvoa T. Alves A. da Costa S. Gonçalves A. (2018). BALB/c mice infected with DENV-2 strain 66985 by the intravenous route display injury in the central nervous system. *Sci. Rep.* 8:9754. 10.1038/s41598-018-28137-y 29950590 PMC6021404

[B195] Solomon T. Mallewa M. (2001). Dengue and other emerging flaviviruses. *J. Infect.* 42 104–115. 10.1053/jinf.2001.0802 11531316

[B196] Solomon T. Kneen R. Dung N. Khanh V. Thuy T. Ha D. (1998). Poliomyelitis-like illness due to Japanese encephalitis virus. *Lancet.* 351 1094–1097. 10.1016/S0140-6736(97)07509-7500 9660579

[B197] Solomon T. Ni H. Beasley D. Ekkelenkamp M. Cardosa M. Barrett A. (2003). Origin and Evolution of Japanese Encephalitis Virus in Southeast Asia. *J. Gen. Virol.* 77 3091–3098. 10.1128/JVI.77.5.3091-3098.2003 12584335 PMC149749

[B198] Sountharalingam S. Herath H. M. Wijegunasinghe D. Senanayke S. (2017). Opsoclonus myoclonus syndrome in a patient with Japanese encephalitis: a case report. *J. Med. Case Rep.* 11 1–4. 10.1186/s13256-017-1454-1455 29058581 PMC5651589

[B199] Srikiatkhachorn A. Kelley J. F. (2014). Endothelial cells in dengue hemorrhagic fever. *Antiviral. Res.* 109 160–170. 10.1016/j.antiviral.2014.07.005 25025934 PMC4148486

[B200] Sriurairatna S. Bhamarapravati N. Phalavadhtana O. (1973). ). Dengue virus infection of mice: morphology and morphogenesis of dengue type-2 virus in suckling mouse neurons. *Infection. and Immunity.* 8 1017–1028. 4594115 10.1128/iai.8.6.1017-1028.1973 PMC422964

[B201] St John A. Rathore A. Raghavan B. Ng M. Abraham S. (2013). Contributions of mast cells and vasoactive products, leukotrienes and chymase to dengue virus-induced vascular leakage. *eLife* 2:e00481. 10.7554/eLife.00481 23638300 PMC3639510

[B202] Su H. L. Lin Y. L. Yu H. P. Tsao C. H. Chen L. K. Liu Y. T. (2001). The effect of human bcl-2 and bcl-X genes on dengue virus-induced apoptosis in cultured cells. *Virology* 282 141–153. 10.1006/viro.2000.0820 11259197

[B203] Suh H. Brosnan C. Lee S. (2009). Toll-like receptors in CNS viral infections. *Curr. Trop. Microbiol. Immunol.* 336 63–81. 10.1007/978-3-642-00549-7_4 19688328

[B204] Suman V. Roy U. Panwar A. Raizada A. (2016). Japanese Encephalitis complicated with obstructive hydrocephalus. *J. Clin. Diagn. Res.* 10 OD18–OD20. 10.7860/JCDR/2016/16917.7274 27042509 PMC4800575

[B205] Suwanprinya L. Morales N. Sanvarinda P. Dieng H. Okabayashi T. Morales-Vargas R. (2017). Dengue virus-induced reactive oxigen species production in rat microglial cells. *Jpn. J. Infect. Dis.* 70 383–387. 10.7883/yoken.JJID.2016.236 28003593

[B206] Swarup V. Ghosh J. Das S. Basu A. (2008). Tumor necrosis factor receptor-associated death domain mediated neuronal death contributes to the glial activation and subsequent neuroinflammation in Japanese encephalitis. *Neurochem. Int.* 52 1310–1321. 10.1016/j.neuint.2008.01.014 18325634

[B207] Szretter K. Samuel M. Gilfillan S. Fuchs A. Colonna M. Diamond M. (2009). The immune adaptor molecule SARM modulates tumor necrosis factor alpha production and microglia activation in the brainstem and restricts West Nile Virus pathogenesis. *J. Virol.* 83 9329–9338. 10.1128/JVI.00836-839 19587044 PMC2738257

[B208] Tan I. Smith B. von Geldern G. Matten F. McArthur J. (2012). HIV-associated opportunistic infections of the CNS. *Lancet Neurol.* 11 605–617. 10.1016/S1474-4422(12)70098-70094 22710754

[B209] Thongtan T. Cheepsunthorn P. Chaiworakul V. Rattanarungsan C. Wikan N. Smith D. (2010). Highly permissive infection of microglial cells by Japanese encephalitis virus: a possible role as a viral reservoir. *Microbes Infec.* 12 37–45. 10.1016/j.micinf.2009.09.013 19786116

[B210] Thongtan T. Wikan N. Wintachai P. Rattanarungsan C. Srisomsap C. Cheepsunthorn P. (2012). Characterization of putative japanese encephalitis virus receptors molecules on microglial cells. *J. Med. Virol.* 84 615–623. 10.1002/jmv.23248 22337301

[B211] Tohidpour A. Morgun A. V. Boitsova E. B. Malinovskaya N. A. Martynova G. P. Khilazheva E. D. (2017). Neuroinflammation and infection: molecular mechanisms associated with dysfunction of neurovascular unit. *Front. Cell. Infect. Microbiol.* 7:276. 10.3389/fcimb.2017.00276 28676848 PMC5476750

[B212] Torti N. Walton S. Brocker T. Rülicke T. Oxenius A. (2011). Non-hematopoietic cells in lymph nodes drive memory CD8 T cell inflation during murine cytomegalovirus infection. *PLoS Pathog.* 7:e1002313. 10.1371/journal.ppat.1002313 22046127 PMC3203160

[B213] Trung D. Wills B. (2010). Systemic vascular leakage associated with dengue infections the clinical perspective. *Curr. Top. Microbiol. Immunol.* 338 57–66. 10.1007/978-3-642-02215-9_5 19802578

[B214] Tsai T. Chen C. Lin Y. Chang C. Tsai C. Cheng Y. (2016). Microglial retard dengue virus induce acute viral encephalitis. *Sci. Rep.* 6:27670 10.1038/sresp27670 PMC4899773 27279150

[B215] Tung W. H. Tsai H. W. Lee I. T. Hsieh H. L. Chen W. J. Chen Y. L. (2010). Japanese encephalitis virus induces matrix metalloproteinase-9 in rat brain astrocytes via NF-kB signalling dependent on MAPKs and reactive oxygen species. *Br. J. Pharmacol.* 161 1566–1583. 10.1111/j.1476-5381.2010.00982.x 20698853 PMC3010568

[B216] van Cleef K. Overheul G. Thomassen M. Kaptein S. Davidson A. Jacobs M. (2013). Identification of a new dengue virus inhibitor that targets the viral NS4B protein and restricts genomic RNA replication. *Antiviral. Res.* 99 165–171. 10.1016/j.antiviral.2013.05.011 23735301

[B217] van den Pol A. Mao G. Yang Y. Ornaghi S. Davis J. (2017). Zika virus targeting in the developing brain. *J. Neurosci.* 37 2161–2175. 10.1523/JNEUROSCI.3124-16.2017 28123079 PMC5338758

[B218] van der Schaar H. M. Rust M. J. Chen C. Ende-Metselaar H. V. Wilschut J. Zhuang X. (2008). Dissecting the cell entry pathway of dengue virus by single-particle tracking in living cells. *PLoS Pathog.* 4:e1000244. 10.1371/journal.ppat.1000244 19096510 PMC2592694

[B219] van Kralingen C. Kho D. Costa J. Angel C. Graham E. (2013). Exposure to inflammatory cytokines IL-1β and TNFα induces compromise and death of astrocytes. implications for chronic neuroinflammation. *PLoS One* 8:e84269. 10.1371/journal.pone.0084269 24367648 PMC3868583

[B220] van Marle G. Antony J. Ostermann H. Dunham C. Hunt T. Halliday W. (2007). West Nile virus-induced neuroinflammation: glial infection and capsid protein-mediated neurovirulence. *J. Virol.* 81 10933–10949. 10.1128/jvi.02422-06 17670819 PMC2045515

[B221] Vásquez M. García J. Gutiérrez B. Santos L. Villegas N. Cedillo L. (2009). A clinical isolate of dengue virus and its proteins induce apoptosis in HMEC-1 cells: a possible implication in pathogenesis. *Arch. Virol.* 154 919–928. 10.1007/s00705-009-0396-7 19440830

[B222] Velandia M. Castellanos J. E. (2012). “Flavivirus neurotropism, neuroinvasion, neurovirulence and neurosusceptibility clues to understanding flavivirus- and dengue-induced encephalitis,” in *Viral Genomes - Molecular Structure, Diversity, Gene Expression Mechanisms and Host- Virus Interactions*, eds Garciìa M. L. Romanowski V. (Croacia: InTech), 219–240.

[B223] Velandia-Romero M. L. Acosta-Losada O. Castellanos J. E. (2012). In vivo infection by a neuroinvasive neurovirulent dengue virus. *J. Neurovirol.* 18 374–387. 10.1007/s13365-012-0117-y 22825914

[B224] Velandia-Romero M. Calderón-Peláez M. A. Castellanos J. (2016). In vitro infection with dengue virus induces changes in the structure and function of the mouse brain endothelium. *PLoS One.* 11:e0157786. 10.1371/journal.pone.0157786 27336851 PMC4919088

[B225] Verkhratsky A. Matteoli M. Parpura V. Mothet J. Zorec R. (2016). Astrocytes as secretory cells of the central nervous system: idiosyncrasies of vesicular secretion. *EMBO J.* 35 239–257. 10.15252/embj.201592705 26758544 PMC4741299

[B226] Verma S. Kumar M. Nerurkar V. (2011). Cyclooxygenase-2 inhibitor blocks the production of West Nile virus-induced neuroinflammatory markers in astrocytes. *J. Gen. Virol.* 92 507–515. 10.1099/vir.0.026716-26710 21106803 PMC3081232

[B227] Verma S. Kumar M. Gurjav U. Lum S. Nerurkar V. (2010). Reversal of West Nile virus-induced blood-brain barrier disruption and tight junction proteins degradation by matrix metalloproteinases inhibitor. *Virology.* 397 130–138. 10.1016/j.virol.2009.10.036 19922973 PMC3102050

[B228] Wan S. W. Lin C. F. Lu Y. T. Lei H. Y. Anderson R. Lin Y. S. (2012). Endothelial cell surface expression of protein disulfide isomerase activates β1 and β3 integrins and facilitates dengue virus infection. *J. Cell Biochem.* 113 1681–1691. 10.1002/jcb.24037 22422622

[B229] Wang J. Liu J. Zhou R. Ding X. Zhang Q. Zhang C. (2018). Zika virus infected primary microglia impairs NPCs proliferation and differentiation. *Biochem. Biophys. Res. Commun.* 497 619–625. 10.1016/j.bbrc.2018.02.118 29453985

[B230] Wei H. Y. Jiang L. F. Fang D. Y. Guo H. Y. (2003). Dengue virus type 2 infects human endothelial cells through binding of the viral envelope glycoprotein to cell surface polypeptides. *J. Gen. Virol.* 84 3095–3098. 10.1099/vir.0.19308-19300 14573814

[B231] White R. Jakeman L. (2008). Don’t fence me in: harnessing the beneficial roles of astrocytes for spinal cord repair. *Rest Neurol. Neuroscience.* 26 197–214. PMC2825119 18820411

[B232] Wilder-Smith A. Ooi E. E. Horstick O. Willis B. (2019). Dengue. *Lancet* 393 350–363. 10.1016/S0140-6736(18)32560-1 30696575

[B233] Wilson M. Zimmermann L. Crawford E. Sample H. Soni P. Baker A. (2017). Acute west nile virus meningoencephalitis diagnosed via metagenomic deep sequencing of cerebrospinal fluid in a renal transplant patient. *Am. J. Transplant.* 17 803–808. 10.1111/ajt.14058 27647685 PMC5347949

[B234] Winkelmann E. R. Luo H. Wang T. (2016). West nile virus infection in the central nervous system. *F1000Res* 5:F1000FacultyRev–105. 10.12688/f1000research.7404.1 26918172 PMC4755400

[B235] Withana M. Rodrigo C. Chang T. Karunanayake P. Rajapakse S. (2014). Dengue fever presenting with acute cerebellitis: a case report. *BMC Res. Notes* 7:125. 10.1186/1756-0500-7-125 24598036 PMC3973998

[B236] World Health Organization [WHO] (2009). *Dengue Guidelines for Diagnosis, Treatment, Prevention and Control New Edition*. Geneva: World Health Organization Press, 147. 23762963

[B237] Wu-Hsieh B. Yen Y. Chen H. (2009). Dengue hemorrhage in a mouse model. *Ann. N. Y. Acad. Sci.* 1171 E42–E47. 10.1111/j.1749-6632.2009.05053.x 19751401

[B238] Xiang J. Zhang Y. Tan Z. Huang J. Zhao Y. (2014). Guillain-Barré syndrome associated with Japanese encephalitis virus infection in China. *Viral. Immunol.* 27 418–429. 10.1089/vim.2014.0049 25140441

[B239] Yamakazi T. Mukouyama Y. (2018). Tissue specific origin. development, and pathological perspectives of pericytes. *Front. Cardiovasc. Med.* 5:78. 10.3389/fcvm.2018.00078 29998128 PMC6030356

[B240] Yam-Puc J. C. Cedillo-Barrón L. Aguilar-Medina E. M. Ramos-Payán R. Escobar-Gutiérrez A. Flores-Romo L. (2016). The cellular bases of antibody responses during dengue virus infection. *Front. Immunol.* 7:218. 10.3389/fimmu.2016.00218 27375618 PMC4893500

[B241] Yang C. M. Lin C. C. Lee I. T. Yang C. M. Chen W. J. Jou M. J. (2012). Japanese encephalitis virus induces matrix metalloproteinase-9 expression via a ROS/c-Src/ PDGFR/PI3K/Akt/MAPKs-dependent AP-1 pathway in rat brain astrocytes. *J. Neuroinflammation* 9:12. 10.1186/1742-2094-9-12 22251375 PMC3298505

[B242] Yang J. Zou L. Yang Y. Yuan J. Hu Z. Liu H. (2016). Superficial vimentin mediates DENV-2 infection of vascular endothelial cells. *Sci. Rep.* 6 1–12. 10.1038/srep38372 27910934 PMC5133558

[B243] Yang L. Kress B. Weber H. Thiyagarajan M. Wang B. Deane R. (2013). Evaluating glymphatic pathway function utilizing clinically relevant intrathecal infusion of CSF tracer. *J. Transl. Med.* 11:107. 10.1186/1479-5876-11-107 23635358 PMC3665671

[B244] Yauch L. Sheresta S. (2008). Mouse models of dengue virus infection and disease. *Antiviral. Res.* 80 87–93. 10.1016/j.antiviral.2008.06.010 18619493 PMC3048811

[B245] Yeo P. Pinheiro L. Tong P. Lim P. Sitoh Y. (2005). Hippocampal involvement in dengue fever. *Singapore Med. J.* 46 647–650. 16228099

[B246] Zaltzman R. Klein C. Gordon C. (2017). Opsoclonus myoclonus ataxia associated with West Nile virus infection: a dramatic presentation with benign prognosis? *J. Neurol. Sci.* 376 38–41. 10.1016/j.jns.2017.02.057 28431624

[B247] Zhang J. Wang J. Gao N. Chen Z. T. Tian Y. P. An J. (2007). Up-regulated expression of β3 integrin induced by dengue virus serotype 2 infection associated with virus entry into human dermal microvascular endothelial cells. *Biochem. Biophys. Res. Commun.* 356 763–768. 10.1016/j.bbrc.2007.03.051 17382900

[B248] Zhang Y. L. Ouyang Y. B. Liu L. G. Chen D. X. (2015). Blood-brain barrier and neuro-AIDS. *Eur. Rev. Med. Pharmacol. Sci.* 19 4927–4939. 26744885

[B249] Zhao Z. Nelson A. R. Betsholtz C. Zlokovic B. V. (2015). Establishment and dysfunction of the blood-brain barrier. *Cell* 163 1064–1078. 10.1016/j.cell.2015.10.067 26590417 PMC4655822

[B250] Zompi S. Harris E. (2012). Animal models of dengue virus infection. *Viruses.* 4 62–82. 10.3390/v4010062 22355452 PMC3280519

[B251] Zorec R. Županc T. Verkhratsky A. (2018). Astrogliopathology in the infectious insults of the brain. *Neurosci. Lett.* 689 56–62. 10.1016/j.neulet.2018.08.003 30096375

