# Supplementary material for: Dengue Virus Infection of Blood–Brain Barrier Cells: Consequences of Severe Disease
Source: Front Microbiol. 2019 Jun 26;10:1435. doi: 10.3389/fmicb.2019.01435 (PMC6606788; doi:10.3389/fmicb.2019.01435)
Supplement: Supplementary file 1 [file Table_1.docx]

**Supplementary Material**

**Supplemental table S1: Clinical reports of patients with neurological symptoms previously infected with JEV, WNV or ZIKV.**

| **JEV** | **Patients/ Age (Years)** | **Viral Symptoms** | **Neurologic Symptoms** | **(+) Lab tests** | | **Molecular and Other Tests** | **Dx** | **Outcome (number of cases)** | **Ref** |
| --- | --- | --- | --- | --- | --- | --- | --- | --- | --- |
|  |  |  |  | **Serum** | **CSF** |  |  |  |  |
|  | 5 pregnant women (20-35) | Loss of conscious-ness, pyrexia, convulsions. | Clonic rigidity, hemiplegia. | IgM |  | **BTB:** JEV isolation  (1 fetus) | TT | L (2),  CR (2), Mothers: CR | Chaturvedy et al., 1980. |
|  | 12 CH,  M (22) | Fever and headache | (↓) Reflexes, flaccid tone, muscle weakness, (↓) AP of motor neurons. | IgM | IgM IgG | **EMG** and **NCV**: Loss of muscle mass.  **TEM:** Motor axonopathy and/or disorder of the anterior horn cells | PLFP | L (3), RWS (10) | Solomon et al., 1998.  Chung et al., 2007. |
|  |  |  |  |  |  |  | SFP |  |  |
|  | M (6-60)  F (11;26)  4 CH (0,7 ;6) | Fever, nausea, vomiting, drowsiness, headache, myalgia, maculo-papular rash. | Confusion, rigidity, somnolence, sialorrhea, tremors, myoclonus, photophobia, (↓) consciousness, seizures, peripheral nerves palsy, right hemiparesis. | IgM | IgM | **IF** in CSF: JEV (+).  **MRI:** Extensive patchy lessons. **PRNT**: JEV (+). | ENC | RWS (1)  CR (5),  L (1) | Lehtinen et al., 2008.  CDC. 2011.  Jia et al., 2011.  Langevin et al., 2012.  Doti et al., 2013.  Cheng et al., 2018. |
|  |  |  |  |  |  |  | ND |  |  |
|  | M (23) | Fever and headache | Weakness, numbness, facial nerve paralysis, sensory disturbances. | IgM | IgM | **EMG:** Acute demyelinating and sensorimotor polyradiculoneu-ropathy of the limbs. | GBS | CR | Xiang et al., 2014. |
|  | F (36) | Fever and headache | Asymmetric rigidity, plantar reflex bilaterally equivocal. | IgM | IgM | **MRI:** HENC | OH | RWS | Suman et al., 2016. |
|  | F (18) | Fever, headache, vomiting. | Double vision, weakness, rectus palsy, neck stiffness, signs of meningeal irritation. |  | IgM | **MRI:** Hyperintensities in bilateral thalami and limbic region. | LM | L | Narayanan et al., 2017. |
|  | F (31) | Fever, (↓) con-sciousness, headache and myalgia. | Involuntary twitching movements, lower limbs weakness. |  | IgM | **EEG:** Epileptiform discharges, left-sided lateralization | OMS | RWS | Sountharalingam et al., 2017. |
|  | M (35) | Fever, tonic-clonic convul-sions. | Generalized hypotonia, weakness and areflexia, altered sensorium. | IgM | IgM | **MRI:** ENC | TM | L | Mohta et al., 2017 |
| WNV | 3 CH  (4-14)  8 adults (29-87)  M (38-75)  F (75) | Fever, rash, headache, diarrhea, vomiting, chills, dizziness. | Confusion, weakness, (↓) deep tendon reflexes, paresis, paralysis, somnolence, aphasia, limb spasticity, facial nerve paralysis, tremors, memory loss. | IgM | IgM | **BTB**: HENC, microglial nods, reactive gliosis, neuronal loss, necrosis, edema, perivascular infiltrate. **EMG/NCV:** AP. **TEM:** Viral particles in neurons. **MRI:** Myelitis and arachnoiditis  **PRNT:** (+) | ENC | L (8), RWS (6) CR (1) | George et al., 1984.  Asnis et al., 2000.  Giladi et al., 2001.  Cushing et al., 2004.  Hwang et al., 2015. |
|  |  |  |  |  |  |  | MGT |  |  |
|  | 8 adults (20-90) | Fever, weakness, headache, myalgia, arthralgia, anorexia, diarrhea | Altered mental status. |  | IgM | **BTB** and **PCR:** WNV (+). **IMH**: Microglial nods with neuronal degeneration | ENC  (6 C) | L (5)  RWS (3) | Sampson et al., 2001.  Iwamoto et al., 2003. |
|  |  |  |  |  |  |  | MGEN  (2 C) |  |  |
|  | M (70-81):  F (74) | Fever, nausea, vomiting, ataxia, tachycardia | Confusion, lethargy, rigidity, tremors, flaccid paralysis, tonic-clonic seizures, deep coma, no cranial nerve responses, no response to stimulation, absence of deep tendon reflexes. | IgM IgG | IgM | **MRI:** Abnormal meninges and cauda equina enhancement **IMH:** Inflammatory infiltrate, viral antigens in neurons.  **EEG**: Diffuse ENP. **EMG/NCV**: Severe loss of the motor and sensory nerve fibers and diffuse denervation | MGEN | L | Kelley et al., 2003.  Doron et al., 2003. |
|  |  |  |  |  |  |  | PLFP |  |  |
|  | M (40-43) | Nausea, vomiting, diarrhea, fever, confusion, chills, headache and myalgia | Confusion, non-directed flexion responses to deep pain, absent gag reflex, and sluggish. | IgM | IgG | **BTB:** ENC with edema, hemorrhage, and neuronal necrosis | ENC | L (1),  CR (1) | DeSalvo et al., 2004. |
|  | M (50) | Fever, right upper limb weakness | Loss of consciousness. | IgM | IgM | **IMH**: Microglial nods, immune cells cuffing, neuronophagia. **PCR:** WNV (+), **Virus isolation** (+) | MGEN | L | Reddy et al., 2004. |
|  | M (42- 69)  F (61) | Fever, vomiting, headache, hand tremor, muscle weakness, bloody diarrhea, anorexia, chills. | Altered mental status, neck rigidity, bilateral K/B, paralysis, areflexia, opsoclonus, truncal ataxia and tremor | IgM IgG | IgM IgG | Viral isolation and sequencing (+) **Lab tests:** Disseminated intravascular coagulation.  **MRI:** Microangio-pathic lesions | PF | L (2)  CR (1) | Baymakova et al., 2016.  Shaha et al., 2016.  Zaltzman et al., 2017. |
|  |  |  |  |  |  |  | OMS ataxia |  |  |
|  | F (14);  M (69) | Fever, chills, neck and back pain, respiratory illness | Headaches, agitation, confusion, weakness, paresthesia, unsteadiness of gait, language impairment, unable to follow commands. | IgM | IgM | **MRI:** Edema, lepto-meningeal brainstem and cervical spine enhancement. **BTB:** Inflamma-tory neuropathy, myelin damage and axonal degeneration | Acute ENM | RWS | Wilson et al., 2017.  Al-Fifi et al., 2018. |
|  |  |  |  |  |  |  | Acute DNP |  |  |
|  | M (63) | Lumbar pain, fever, nauseas, vomiting | Neurogenic bladder, and bowel, flaccid and areflexic paralysis | IgM |  | **MRI**: Mild swelling of the lumbar spine and abnormal conus medullaris | ES | RWS | Hawkes et al., 2018. |
| ZIKV | M (22-78); F (49) | Limb numbness, unsteady gait, leg swelling, erythematous and pustular lesions, joint pain and myalgia | (↓) Limbs power, areflexia, vibratory feet sensation. Loss of deep tendon reflexes. Paresthesia, fatigue, diplopia, dysphagia, facial nerve palsy, ataxia. | IgM IgG | IgM | **RT-PCR** ZIKV (+). **NCV, EMG and EDS:** Demyelinating polyneuropathy. **PRNT:** ZIKV (+) | GBS | RWS (3)  L (1) | Fabrizius et al., 2016.  do Rosario et al., 2016.  Siu et al., 2017.  Dirlikov et al., 2018. |
|  | M  (45-62) | Headache, arthralgia, asthenia, fever, diarrhea | Weakness of the limbs, areflexia, ptosis of the left eye, diplopia. |  |  | **RT-PCR:** Serum (+). **NCV:** Defect in neuromuscular transmission | MG | RWS | Molko et al., 2017. |
|  | M (36) | Fever, headache, seizures | Left-sided hemiplegia, confusion, hallucinations. |  |  | **RT-PCR:** CSF (+). **MRI:** Low cerebral blood flow, edema. | ENC | L | Schwartzmann et al., 2017. |
|  | F (9-24) | Distal pain, weakness, hyporeflexia | Burning pain in hands and feet, paresthesia in hands and feet, numbness. |  |  | **Nerve ultrasound:** Bilaterally enlarged median nerves. **RT-PCR:** CSF and serum (+) | Acute PN | CR | Nascimento et al., 2017. |
|  | F (36) | Fever, fatigue, headaches, and rash | Confusion, neck stiffness, seizures, motor neuron dysfunction | IgM | IgM | **RT-PCR**: Urine (+) **MRI:** MGEN | MGEN | RWS | Pradhan et al., 2017. |
|  |  |  |  |  |  |  | TM |  |  |
|  | F (24) | Vomiting, fever, myalgia epigastric pain | Bilateral lower limbs weakness, flaccid quadriplegia and confusion | IgG |  | **PRNT:** Zika (+); **NCV and EMG:** GBS | GBS | RWS | Mancera-Paez et al., 2018. |
|  |  |  |  |  |  |  | ENM |  |  |

**AP**: axonal polyneuropathy; **BTB**: Brain tissue biopsy; **C**: cases; **CH**: children; **CSF:** cerebrospinal fluid **CR**: complete recovery; **DNP**: demyelinating neuropathy; **Dx**: diagnosis; **ENC**: encephalitis; **EDS**: Electrodiagnostic studies; **EEG**: electroencephalogram; **EMG**: electromyography; **ENM**: Encephalomielitis; **ES:** Elsberg syndrome; **F**: female; **GBS:** Guillain Barré Syndrome; **HENC:** hemorrhagic encephalitis; **IF**: immunofluorescence; **IMH**: immunohistochemical analysis; **K/B**: Kerning and Brudzinski test; **L**: lethal; **LM:** longitudinal myelitis; **M**: male; **MG:** Myastenia gravis; **MGT**: meningitis; **MGEN:** Meningoencephalitis; **MRI:** Magnetic resonance imaging; **NCV**: Nerve conduction studies; **ND**: Neurologic deficit; **OH:** obstructive hydrocephalus; **OMS**: opsoclonus myoclonus syndrome; **PCR:** polimerase chain reaction; **PF:** Purpura fulminans; **PN**: polyneuritis; **PLFP**: Polio-like Flaccid Paralysis; **PRNT**: plaque reduction neutralization test; **RT-PCR:** Polymerase chain reaction with retro-transcription; **RWS**: recovered with sequelae; **SFP:** Severe flaccid paralysis; **TEM**: transmission electron microscopy; **TM**: Transverse myelitis; **TT:** transplacental virus transmission; **(+)**: positive; **(-)** negative; **(↓)** decrease;

**Supplemental table S2: In vivo/ Ex vivo evidence of JEV, WNV and ZIKV infection in the different BBB cells in human and other models.**

| **JEV** | **Model/ Age/ Tissue** | **Infection Tests, histological and other findings** | **Infection** | **Cellular findings** | **Ref** |
| --- | --- | --- | --- | --- | --- |
|  | Fatal cases 2 adults and 2 children Brain tissue | **Serum and CSF:** IgM and IgG+. **JEV antigen:** medulla, midbrain, left cerebellum, temporal and frontal cortex | **N:** Yes  **EC**: Yes | **N**: JEV antigen in cell bodies processes (probably axons).  **A:** Reactive astrocytes (GFAP+) and clasmatodendrosis.  **M:** MHC II+ in the brain parenchyma. | German et al., 2006. |
|  |  | Swollen brain, microhemorrhages, inflammatory infiltrates, necrosis, vascular congestion, microglial nodule formation. |  |  |  |
|  | Mice (infected with 2 JEV strains)  3-4 weeks old.  Brain tissue | Perivascular cuffs, hemorrhage, cellular infiltrates, mild vascular damage. Necrosis, degenerate neurons and glial cells. Presence of necrotic foci. | NR | **M:** diffuse infiltration and activation, presence of nodes.  **A:** Activation.  **N:** Cell damage |  |
|  | Balb/C mice (infected i.c)  4 days old Brain tissue | RT-PCR + | NR | **N:**(↓) healthy cells.  **A:** Activated (↑) GFAP, GLAST, GLT-1, NGF, ROS and Bax. | Mishra et al., 2007. |
|  |  | Paralysis, poor pain response and body tremor. |  |  |  |
|  | Balb/C mice (infected i.c).  3-4 days old  Brain tissue | High increase in IP-10 expression at 4 dpi.  Cytokines and IFN-γ increased | NR | **M** and **N:** Did not express IP-10.  **A:** Presumed responsible for the (↑) IP-10 | Bhowmick S et al., 2007. |
|  | Balb/C mice (infected i.c)  3-4 days old Brain tissue | Clinical signs: Paralysis, poor pain response and whole-body tremor. | NR | **N:** Apoptosis | Swarup et al., 2008. |
|  |  |  |  | **A:** Astrogliosis and (↑) GLAST-1. |  |
|  |  |  |  | **M:** Evidence of activation. |  |
|  |  | Presence of many pyknotic cells in all areas of the brain. All effects diminish when TRADD was silenced using siRNA |  | **SR:** (↑) of: IL-6, IL-12, TNF-α, IFN-γ, MCP-1, CCR-1, CCR-2, CXCR-3, MMP-9, VCAM-1 and ICAM-1. TIM-1 (↓) |  |
|  | Balb/C mice (infected i.c)  3-4 days old Brain tissue | Limb paralysis, poor pain response and whole-body tremor | NR | **A** and **M**: (↑) IL-1β and IL-18.  **SR**. IL-18 and IL-1β (↑ time-dependent manner). | Das et al., 2008. |
|  | Rhesus macaques (n=12)  3-7 years old  Brain tissue | Mild to moderate, multifocal to diffuse, non-suppurative meningoencephalomyelitis  Tomb-stone like luminal protrusion of EC, vascular damage, perivascular haemorrhage and substantial leakage of serum into the parenchyma. Apoptosis molecules: caspases-8, -9 and cleaved caspase-3 | **N:** Yes **EC:** Yes **A:** No | **N:** Apoptosis, (↑) MMP-9 **EC**: Activated and expressing MHC-II.  **A:** Activated and reactive. **M:** Activated and recruited to the zone of damage, satellitosis.  **A and M:** (↑) Caspase-9, TNF-α, inducible NOS, nitrotyrosine and MMP-2 | Myint et al., 2014. |
| **WNV** | Human Fatal cases 2 adults Brain tissue | RT-PCR: viral genome in brain and spinal cord | **N:** Yes **A:** Yes **M:** Yes | Astrocytes activation | van Marle et al., 2007. |
|  |  | CCL2, CXCL10, IL-1β, and IDO (-) |  |  |  |
|  | KO SARM mice (i.c infected)  8-12-week-old  Brain tissue | IgG and IgM + | **N:** Yes **A:** Yes | **N:** (↑) apoptotic cells.  **M:** (↓) activation | Szretter et al., 2009. |
|  |  | (↓) TNF-α protein. TNF-α gene expression in the brainstem, but not in the cortex |  |  |  |
|  | Swiss Webster mice  5-6-day old Spinal cord slice culture | RT-PCR: Viral replication over time | **N:** Yes (90%) | **A** and **M**: activated  (↑) CXCL10, CCL5, CXCL1, CCL2 IL-6, TNF-α, and TRAI (↓). | Quick et al., 2014. |
|  |  | Large microglial cells in the anterior horn region. | **A:** Yes (11%). **M:** No (<1%) |  |  |
|  | Fatal cases  Brian tissue | Axonal swelling and loss of tubulin. Diameter of the DRG (↑) and these cells appeared enlarged | NR | **A**: S100B immunoreactivity visible in infected tissue | Kuwar et al., 2015. |
|  | Horses n=12, infected i.c. Different ages. Thalamus and hindbrain | Encephalitis: paresis, ataxia, fasciculations, hyperaesthesia | NR | **M:** glial nodes especially in the hindbrain.  **A:** (↑) GFAP+ cells in the hindbrain. | Delcambre et al., 2017. |
|  |  | Severe gliosis, perivascular cuffing and glial nodules |  |  |  |
|  | C57BL/6J 5–8 week-old  Brain tissue | IL-1β (-) during acute infection of neurogenic zones of the CNS favoring astrocyte genesis.  IL-1R1-deficient mice were protected from decreased neurogenesis | NR | **A**: primary source of IL-1β in the recovering hippocampus.  Preferential generation of proinflammatory astrocytes impairs neuronal progenitor cell homeostasis via expression of IL-1 | Garber et al., 2018. |
| **ZIKV** | Organotypic cultures Human Brain tissue | Immunostaining of the viral RNA-NS5 | **N:** More or less  **A:** Yes **M:** Yes | **GLIA:** clusters of infected radial glia.  **M:** In phagocytosis of other infected cells | Retallack et al., 2016. |
|  |  | The virus infected both ventricular and outer radial glial cells |  |  |  |
|  | Human Fetuses  20 and 26 Gestational Weeks or post-ovulatory week.  Brain tissue | Plaque assays, IF | **N:** More or less  **A:** Yes  **M:** Yes | **A** and **M**: AXL (↑), major ZIKV targets | Meertens et al., 2017. |
|  | Human micro encephalic cases (n=9) 14-24 weeks  Brain Tissue | Cell mix tested by flow cytometry and qRT-PCR | **M:** Yes | **M:** Activated  **N:** Developing cortex neurons death. | Lum et al., 2017. |
|  |  | Microglial activation and virus dissemination into brain parenchyma |  |  |  |
|  | Mice C57BL/6J | TCID50 assay in the developing brain | **NPC:** Yes | **N**: cell death and neuronal reduction. | Shao et al., 2017. |
|  | Embryos (E0.5)  Brain tissue | African ZIKV isolate is more potent at causing brain damage and postnatal lethality | **N:** Yes | **M**: Cells activated.  **A**: Astrogliosis |  |
|  | White Swiss Mice 0-1-day old Brain tissue | Lethargy, ataxia, paralysis, and running movement | NR | **N:** Neuronal death mainly in cortex.  **A:** Hyperplasia and hypertrophy with overlapping processes. (↑) GFAP+ | Fernandes et al., 2017. |
|  |  | Neuronal somas shrunken with cytoplasmic eosinophilia and pyknosis or karyolisis. White matter degenerated, with vacuolization. Disruption of cortex and hippocampus architecture |  |  |  |
|  | C57BL/6 mice Neonates (P0)  Brain tissue | IF+ | **N:** Yes | **A**: first type of cells infected by ZIKV | van den Pol et al., 2017. |

**A:** Astrocytes; **DRG:** Dorsal root ganglion; **EC:** Endothelial cells; **i.c:** intra-cerebral; **IF:** immunofluorescence; **KO:** knock out; **M:** Microglia; **N:** Neuron; **n:** number of samples; **NR:** Not reported; **NPC:** Neural progenitor cells; **RT-PCR:** Polymerase chain reaction with retro-transcription; **qRT-PCR**: Quantitative polymerase chain reaction with retro-transcription; **siRNA:** small interfering RNA; **SR:** Systemic response. **(+):** positive; **(-)**: negative; **(↑)**: Increased expression; **(↓)**: Decreased expression.

**Supplemental table S3: In vitro evidence of JEV, WNV and ZIKV infection in the different BBB cells of human and other models.**

| **JEV** | **SPECIE /AGE/ CELL** | **INFECTION** | **CELLULAR FINDINGS** | **REF** |
| --- | --- | --- | --- | --- |
|  | Rats (Sprague-Dawley)  1 day old.  **Neuron-astrocytes cerebral cortex** | **N:** Yes (80%)  **A:** Yes (30-45%) | IL-6, MCP-1 and RANTES (mRNAs)  Lactic acid and glucose uptake | Chen et al., 2000. |
|  | Rats (Sprague-Dawley)  1 day old  **Mixed N/G (microglia and astrocytes), enriched neuron culture** | **N**: Yes (90%), **M**: Yes (80%), **A:** Yes (20-30%) | In N/G cultures RANTES (time-dependent manner).  **N**: undergo a cell death process.  **M** and **A**: not significantly affected by infection | Chen et al., 2004. |
|  | BALB/c and C57BL/6 mice, 1-day-old  **Astrocyte cultures** | Yes | MHC-I (time-dependent manner), Non-classical MHC genes: H-2T23, H-2Q4 and H-2T10t (↑), VCAM-1 (↑) in a MOI independent manner. | Abraham et al., 2006. |
|  | Mouse neuroblastoma cell line **Neuro-2a** | Yes | Production of Nerve Growth Factor (NGF) and ciliary neurotrophin factor (CNTF) from astrocytes, prevent neuronal cell death | Mishra et al., 2007. |
|  | Human cell line of fetal microglial origin **CHME3** | Yes | After infection, microglial cells did not produce high levels of IP-10 | Bhowmick et al., 2007. |
|  | Human fetal astrocyte cell line **SVG** | Yes | Significant increase in IP-10 production |  |
|  | **SVG Cells** | Yes (21,23%) | Prominent CPE, GFAP and MIG (↑), IP-10, MCP-1 and RANTES (↓) | Mishra et al., 2007. |
|  | Human astrocytic cell line: **U87** | Yes (28,45%) | GFAP, IP-10, MCP-1, MIG and RANTES (↑), ROS (↓) |  |
|  | BALB/c and C57BL6,  1 day old  **Astrocyte cultures** | Yes | Non-classical MHC from type 1b (↑) | Abraham et al., 2008. |
|  | **Neuro-2a** | Yes | TRADD (↑) Neuronal loss, diminish when TRADD was silenced using siRNA | Swarup et al., 2008. |
|  | **CHME3 Cells** | **A**: Yes  **M**: Yes | Activation process: **M**+ and **A**+.  Production of IL-18 and IL-1β, albeit at different levels. Cell lines treated with IL-18 and/or IL-1β → IL-8, TNF-α, IL-6, e IP-10, MCP-1, MIG and RANTES (↑). | Daset al., 2008. |
|  | **SVG Cells** |  |  |  |
|  | Human Neuroblastoma cell line **SK-N-SH** | Yes | Supernatants from astrocytes and microglia cells treated with IL-18 and IL-1β → cell apoptosis and caspase 3, cleaved PARP (↑) and Bcl-2 (↓). |  |
|  | Rats (Sprague-Dawley)  1 day old,  **N/G, mixed glia, neuron, microglia, and astrocyte cultures** | **N**: Yes  **M**: Yes  **A**: Yes | Degeneration of neurite processes and (↓) numbers of visible neurons.  **A:** not significantly affected but produced IL-6 and RANTES.  **M:** activated.  Supernatants from mixed glial cells caused neuronal death. TNF-α, IL-1β, IL-6 and RANTES (↑) in N/G cultures, mixed glial cultures and microglial cultures infected. | Chen et al., 2010. |
|  | **RBA-1 cells**,  Astrocyte cell line of neonatal rat cerebrum | Yes | MMP-9 protein expression in a time-dependent manner. Generation of ROS via (↑) NADPH oxidase activity, after activation of p42/p44 MAPK and NF-kB. | Tung et al., 2010. |
|  | Rats (Sprague-Dawley)  1 day old  **N/G, mixed glia, neuron, microglia, and astrocyte cultures** | **N**: Yes  **A**: Yes | RANTES (↑) in Mixed glia, **A**, and **M.** Cytokines TNF-α and IL-1β derived from microglia and astrocytes are involved in triggering RANTES production | Chen et al., 2011a. |
|  |  | **M**: Yes | TNF-α and IL-1β (mRNA and protein) (↑) activity of Src, Ras, and Raf. | Chen et al., 2011b. |
|  | **RBA-1 cells** | Yes | Expression of pro-form MMP-9 via ROS/c-Src/PDGFR/PI3K/Akt/ MAPKs-dependent of AP-1 activation | Yang et al., 2012. |
|  | Rats (Sprague-Dawley)  1 day old  **EC and astrocyte cultures** | **EC:** Yes  **A:** Yes | Infected astrocytes released molecules that activated ubiquitin proteasome, degraded ZO-1 and claudin-5, and disrupted endothelial barrier integrity. Vascular endothelial growth factor (VEGF), IL-6, and MMP-2/MMP-9 (↑) | Chen et al., 2015 |
|  | Human brain endothelial cells: **HBEC. Human astrocytes** | **EC:** Yes  **A**: Yes | EC had a limited viral replication. IL-6, CCL5 and CXCL10 (↑). TEER correlations were found for mediators: IL-6, CXCL10, VCAM, MMP7 and leptin | Patabendige et al., 2018. |
| **WNV** | Mouse Brain  Neonates  **Astrocyte culture**. | Yes | Interferon production and up-regulates class I and II MHC antigen expression | Liu et al., 1998. |
|  | Cells isolated from human fetus.  **Highly enriched neuronal cell, purified astrocyte and microglial cell cultures.** | **N:** Yes (80%)  **A:** Yes (30%)  **M:** More or less (<1%) | **A:** produced CXCL10 and CCL5, **M:** produced IL-6 and TNF-α, CXCL10, CCL2 and CCL5 associated with MAPK pathway | Cheeran et al., 2005. |
|  | Human neuroblastoma cells **LAN-2** | **N:** Yes (8,2%) | Astrocytes overexpressed the CCL2, CXCL10 and IL-1 genes, | van Marle et al., 2007. |
|  | Human glioblastoma astrocytoma: **U373** | **A**: (3,3%) | Neurons expressed CXCL10 and IL-1β. The infection induced apoptosis in a MOI depending manner. |  |
|  | Human brain cortical astrocytes **HBCA** | **EC:** Yes | EC induced overexpression of TIMP, MMP-1, 3 and 9 | Verma et al., 2010. |
|  | Human brain microvascular EC: **HBMVE** | **A**: Yes | The MMPs released by WNV-infected HBCA cells, degrade TJP (ZO-1 and claudin-1) of HBMVE cells |  |
|  | Human neuroblastoma cell line: **SK-N-SH** | Yes | Expression of IL-1β, -6, -8, and TNF-α in a dose- and time dependent manner, coinciding with cell death | Kumar et al., 2010. |
|  | **HBCA** | Yes (79%) | Expression (RNA and protein) of COX-2, PGE2, IL-1β, -6 and -8. All of these were reduced with Cox-2 blocker NS-398, that also attenuated the expression of MMP-1, -3 and -9 | Verma et al., 2011. |
|  | Human brain microvascular EC: **HBMEC** | Yes | Infection effective with a none or a highly pathogenic virus strain. Expression of CCL5 with both strains | Hussmann et al., 2014. |
|  | **HBCA** | Yes | Secreted high levels of CCL5 |  |
|  | Mouse glioma cell line: **GL 261** |  | The UV-WNV-treated cells showed a strong immunoreactivity to S100B.  Astrocytes showed higher immunoreactivity to S100B. UV-WNV particles also caused induction of S100B (mRNA and protein). | Simonin et al., 2016. |
|  | **Primary astrocytes from the cerebral cortex and cerebellum** |  |  |  |
| **ZIKV** | Human primary glioblastoma cell line: **U87** | Yes (<30%) | Human microglia and astrocytes expressed Axl (putative viral receptor) and it mediates ZIKV infection of glial cells | Retallack et al., 2016. |
|  | Human microglial cell line: **CHME3** | **M**: Yes (human, 60% MOI 10), No (murine) | Blocking receptor Axl reduced ZIKV infection of astrocytes. AXL knockdown nearly abolished the infection | Meertens et al., 2017. |
|  | **Primary human and murine astrocytes and microglia.** | **A:** Yes. (Human 60% MOI 10, Murine <8%) |  |  |
|  | Human neural progenitor cells: **hNPCs** | **hNPC:** Yes (>45%) |  |  |
|  | **Human astrocyte cell line** | Yes (43-75%) | Astrogliosis | Simonin et al., 2016. |
|  | Wild-type (WT) and interferon alpha receptor knock out (IFNAR-/-) mice | Yes (<30% WT mice) | Astrocytes control viral replication through a rapid interferon response that restrict viral spread | Lindqviset al., 2016. |
|  | **Primary astrocytes** | Yes (69% IFNAR -/- mice) |  |  |
|  | **Primary human fetal microglial cells** | Yes | (↑) IL-6, TNF-α, IL-1β and MCP-1. | Lum et al., 2017. |
|  | **Normal human astrocytes cell line** | Yes | Large cytoplasmic Paraptosis-like vacuoles | Monel et al., 2017. |
|  | Newborn mice  **Primary microglial cultures** | Yes | TNF-α, IL-6, IL1β and iNOS (↑). | Wang et al., 2018. |
|  | **Neuronal Progenitor Cells: NPC, E14.5 mouse telencephalons** | Yes | Culture of NPCs with ZIKV-microglial conditioned medium impaired the growth of neurospheres and reduced neuronal differentiation of NPC |  |
|  | Mice  Embryonic day 14 (E14) **Mouse neural stem cells** | Yes | Neuronal, astrocytes and oligodendrocyte progenitors (↑ numbers). Infection in astrocytes increased transcription of key genes involved in the antiviral response | Lossia et al., 2018. |
|  | Human umbilical vein endothelial cells: **HUVEC** | Yes | The PKA inhibitor PKI 14-22 (PKI) might be a potent inhibitor of ZIKV replication | Cheng et al., 2018. |
|  | **HUVEC** | Yes | Induction of autophagic response | Peng et al., 2018. |

**N/G:** neuron/glial cells; **N:** Neuron; **A:** Astrocytes; **M:** Microglia; **EC:** Endothelial cells; **(+)**: positive; **(-)**: negative; **(↑)**: Increased; **(↓)**: Decreased; **NPC:** Neural progenitor cells; **IF:** immunofluorescence.
